# Multivariate classification of multichannel long-term electrophysiology data identifies different sleep stages in fruit flies

**DOI:** 10.1126/sciadv.adj4399

**Published:** 2024-02-21

**Authors:** Sridhar R. Jagannathan, Travis Jeans, Matthew N. Van De Poll, Bruno van Swinderen

**Affiliations:** ^1^ Department of Psychology, University of Cambridge, Cambridge, UK.; ^2^ Institute of Neurophysiology, Charité Universitätsmedizin Berlin, Berlin, Germany.; ^3^ Queensland Brain Institute, The University of Queensland, St Lucia, QLD Australia.

## Abstract

Identifying different sleep stages in humans and other mammals has traditionally relied on electroencephalograms. Such an approach is not feasible in certain animals such as invertebrates, although these animals could also be sleeping in stages. Here, we perform long-term multichannel local field potential recordings in the brains of behaving flies undergoing spontaneous sleep bouts. We acquired consistent spatial recordings of local field potentials across multiple flies, allowing us to compare brain activity across awake and sleep periods. Using machine learning, we uncover distinct temporal stages of sleep and explore the associated spatial and spectral features across the fly brain. Further, we analyze the electrophysiological correlates of microbehaviors associated with certain sleep stages. We confirm the existence of a distinct sleep stage associated with rhythmic proboscis extensions and show that spectral features of this sleep-related behavior differ significantly from those associated with the same behavior during wakefulness, indicating a dissociation between behavior and the brain states wherein these behaviors reside.

## Teaser

Brain recordings and machine learning reveal how flies sleep and identify sleep stages associated with microbehaviors.

## INTRODUCTION

Humans spend a third of their life engaged in sleep, wherein they become less responsive to external stimuli. Most animals studied so far, starting from the tiny fruit fly (*1*, *2*) to the large sperm whale (*3*), display extended periods of quiescence, which are now categorized as sleep. Evolutionary conservation of the sleep state in all animals suggests that its benefits outweigh the potential risks and vulnerabilities brought on by losing awareness of one’s external environment. Sleep deprivation has been shown to produce deficits in learning and memory (*4*), immune system malfunction (*5*), and stress regulation (*6*). However, the organization of sleep in relation to its potential functions remains unclear. Different theories have been proposed for functions of sleep including those involving processes such as neuronal plasticity and synaptic downscaling (*7*) and metabolic waste clearance (*8*). However, sleep research methodology is largely driven by research in humans and other mammals so the primary way of classifying sleep states has therefore been using electrophysiological readouts, such as electroencephalography (EEG). By identifying distinct electrical signatures associated with the different stages of sleep, different functional roles have been hypothesized for them. For example, rapid eye movement (REM) sleep in mammals has been proposed to regulate motor learning and memory consolidation (*9*, *10*), while slow wave sleep has been proposed to regulate synaptic strength and cellular homeostasis mechanisms (*11*).

One of the primary challenges for understanding sleep architecture in invertebrates has been developing a capacity to record and assess brain-wide patterns of electrical activity across long time periods that encompass several sleep-wake transitions. In this context, small animals such as the fruit fly *Drosophila melanogaster* present as extremely challenging subjects, although they already potentially provide a wealth of molecular genetic tools to help better understand sleep biology. Previous sleep studies in flies have either recorded just a single local field potential (LFP) channel during spontaneous sleep bouts (*12*–*15*) or from multichannel probes during short (~15 min) bouts of genetically induced sleep (*12*, *16*). In other work, whole-brain calcium imaging in sleep-deprived flies revealed distinct stages of spontaneous sleep (*17*), although these recordings were rarely long enough to display any revealing sleep architecture, and it remains unclear how these different sleep stages might be manifested across the fly brain from the central complex to optic lobes.

Some reasons for the lack of whole-brain or multichannel sleep data in *Drosophila* are technical in nature: (i) It is difficult to perform long-term electrophysiological recordings with multiple electrodes in such small brains, the survival rate is low, and the recording tools used do not yet allow for consistent spatial positioning of multiple electrodes in different flies. (ii) Calcium imaging, on the other hand, which lacks in temporal precision compared to LFPs, does allow for consistent spatial locations of recordings (with image registration tools); however, concerns with photobleaching and phototoxicity have made it difficult to achieve the long-term recordings to acquire spontaneous sleep data. Subsampling provides one solution: For example, in a recent study, 24-hour recordings were conducted by recording for only 1 s after every minute (thus recording for only 1.6% of the overall time) (*18*). However, this subsampling approach might miss important sleep transitions or longer-lasting sleep phenomena. To best compare the brain activity during sleep in flies with similar data from other animals would ideally involve similar readouts akin to a whole-brain EEG, which, in *Drosophila*, would necessarily involve miniaturized multichannel probes such as used previously for visual studies (*16*, *19*) as well induced sleep (*12*) and anesthesia experiments (*20*–*24*). In addition, these recordings would ideally be supplemented by detailed behavioral analysis beyond the simple locomotory determinants that have traditionally defined sleep in flies (*1*, *2*). Mammalian sleep stages involve distinct microbehaviors in addition to electrophysiological correlates (*25*, *26*), and this seems to be true for some invertebrates as well (*27*–*29*).

In this study, we optimized a multichannel LFP recording preparation for *Drosophila* flies, to track long-term neural activity in 16 channels across one hemisphere of the fly brain, in a transect from the retina to the central complex. The flies underwent spontaneous sleep bouts while walking/resting on an air-supported ball and survived long enough to provide 20 hours of data over one day-night cycle. We developed calibration tools to consistently record from similar spatial locations in different flies. We used machine learning–based methods [support vector machines (SVMs) and random forest classifiers] to first investigate the structure of sleep bouts and further explored the spectral features across multiple brain channels. We also used machine learning techniques (pose tracking and identification) to identify fly microbehaviors during these long-term recordings and to determine their potential association with different sleep stages. Together, our analyses identify neural correlates of sleep stages in the fly central brain, associated with rhythmic proboscis extensions (PEs) as a key behavioral feature. We find that the LFP features associated with PEs during wake and sleep are dissimilar, suggesting that a distinct brain state is driving the sleep functions associated with this rhythmic microbehavior.

## RESULTS

### Behavioral analysis of tethered flies during sleep and wake

**Fig. 1.**
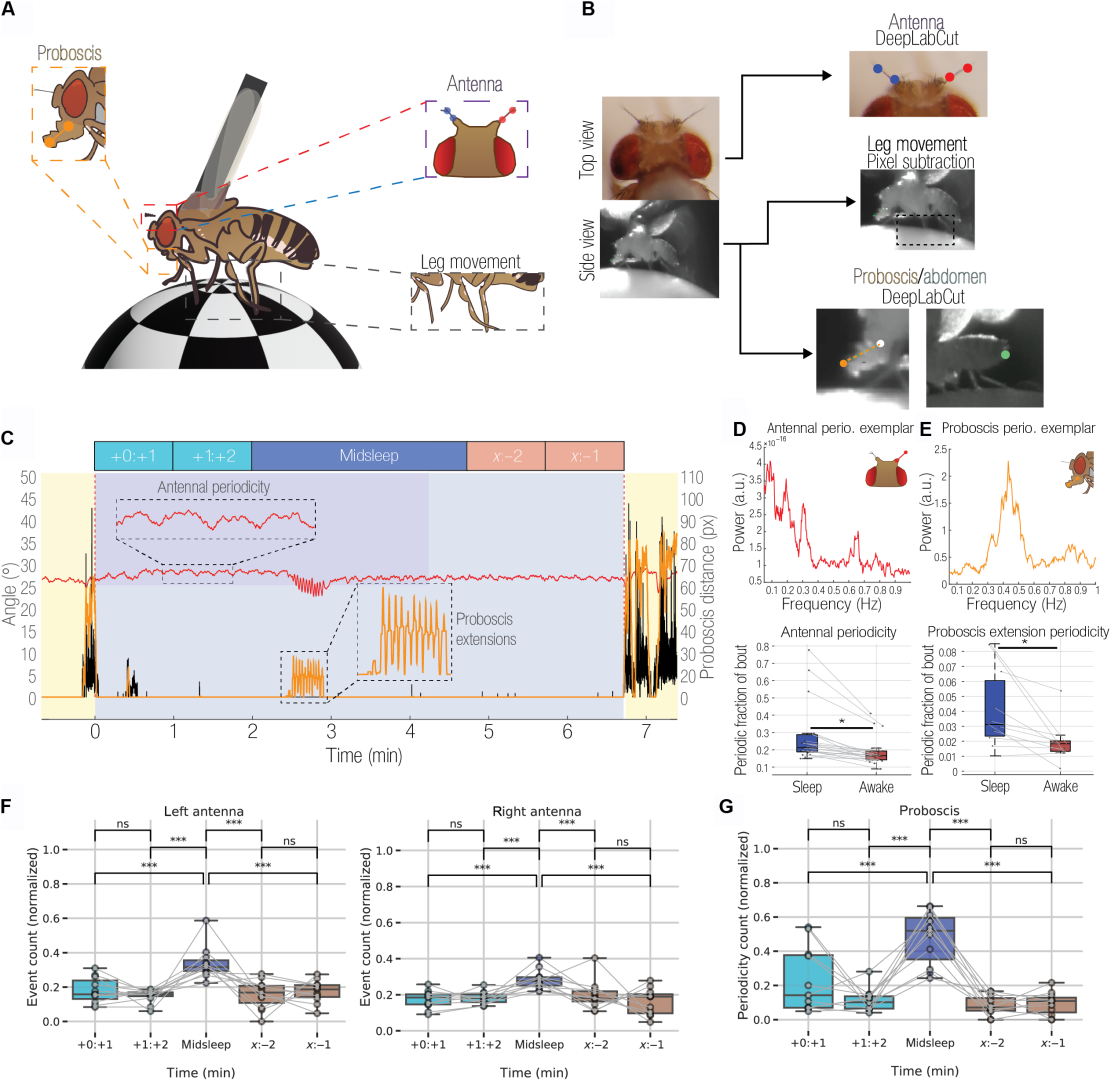
Tethered flies display microbehaviors during sleep. (**A**) Schema for the setup used to record microbehaviors of sleeping and waking flies. A tethered fly walks on an air-supported ball. (**B**) The fly is filmed by two cameras (top view and side view). Footage from these cameras is fed through a preprocessing pipeline that tracks movements of the antennae (top), legs (middle), abdomen, and proboscis (bottom). (**C**) An example sleep bout. Locomotive activity (black) has ceased for long enough (>5 min) that the period of inactivity is classified as a sleep bout. The movement of the right antenna (red trace) shows an apparent low-frequency periodicity (inset) across the 6-min bout, interrupted in the middle by a series of PEs (orange trace; inset). (**D**) Top: Fast Fourier transform of antennal activity during an exemplar sleep bout containing antennal periodicity. Bottom: Comparison of the fraction of sleep and wake that consisted of periodic antennal activity (*P < 0.05, Student’s *t* test). a.u., arbitrary units. (**E**) As with (D), for proboscis periodicity. (**F**) Proportions of antennal periodicity (left and right antennae) across different sleep segments: +0:+1 indicates 1 min after start of sleep, +1:+2 indicates 2 min after start of sleep, *x*:−2 indicates 2 min before end of sleep, and *x*:−1 indicates 1 min before end of sleep. The event count (normalized to total events per epoch) is significantly higher in the midsleep segments compared to other segments. (**G**) As with (F), but for periodic extensions of the proboscis. ***P < 0.001. ns, not significant.

Before conducting any electrophysiological recordings, we first investigated how flies slept when tethered to a rigid metal post while being able to walk on an air-supported ball (Fig. 1A). Flies were filmed overnight under infrared illumination, and locomotory behavior was quantified using a pixel subtraction method (*12*) to identify sleep epochs, defined by the absence of locomotion or grooming behavior for 5 min or more (*1*, *2*, *12*, *13*). We also tracked the movement of different body parts, including the proboscis, antennae, and abdomen to detect potential microbehaviors during sleep. For this, we used machine learning [DeepLabCut (*30*)] to train a classifier to track microbehavioral movements through wake and sleep (Fig. 1B). As shown previously (*12*), tethered flies were able to sleep in this context (Fig. 1C and fig. S1A). Consistent with a previous study (*27*), we also observed regular PEs during sleep bouts (fig. S1B), which often occurred in rhythmic succession (Fig. 1C, orange trace). We also observed antennal movements and found that these were periodic in a subset of flies (Fig. 1C, red trace). Since both antennal movements and PEs were often rhythmic during sleep, we characterized both microbehaviors in the frequency domain (Fig. 1, D and E, top) to determine whether these were different between sleep and wake. We found that a greater proportion of the sleeping states displayed both antennal periodicity and PE periodicity, compared to the waking states (Fig. 1, D and E, bottom; and fig. S1, E and G). However, the time course and presence of individual PEs (fig. S1, B and C) and the dynamics (e.g., inter-PE intervals and frequency) of periodic PEs were not different between sleep and wake (fig. S1, D and F), even if this behavior varied across sleep and wake.

A previous study suggested that PEs during sleep are accomplishing a specific function in flies linked to waste clearance and that these might be specific to a deeper sleep stage (*27*). We therefore next examined whether PE and antennal periodicity varied throughout a sleep bout. For this, we segmented all >5-min sleep bouts into five temporal epochs, as done previously for spontaneous sleep experiments in tethered flies (Fig. 1C, top schema) (*12*, *17*). The first 2 min and last 2 min of sleep (flanked by locomotor behavior) were analyzed separately for microbehaviors and compared to “midsleep” epochs, which could be of different durations. To examine whether the likelihood of periodicity for both antennae and proboscis varied on the basis of the sleep epochs, we used multilevel modeling instead of traditional repeated measures of analysis of variance (ANOVA) (as different flies had varying numbers of sleep epochs). To understand whether the likelihood of the periodicity varies by sleep epoch, we defined two models (separately for both antennae and proboscis): a null model, where the likelihood of periodicity depends on the mean per fly, and an epoch model, where the likelihood of periodicity depends on the epoch (e.g., midsleep, etc.). For details, refer to the “Models for antennal and proboscis periodicity” section. For all the microbehaviors, the “epoch” model (where the periodicity depends only on the sleep epoch) emerged as the winning model, and a reliable main effect of epoch was found (*P* < 0.001) in all cases. Further, we performed post hoc tests using Tukey adjustment (for multiple comparisons) to identify differences between pairs that are significant. Thus, we found an apparent increase in the likelihood of periodicity for both antennae and proboscis during the middle segments of sleep bouts (Fig. 1, F and G). This suggested physiological differences that might be detected in the fly brain, so we then performed electrophysiological recordings in a similar context.

### Long-term multichannel recordings with spontaneous sleep bouts

**Fig. 2.**
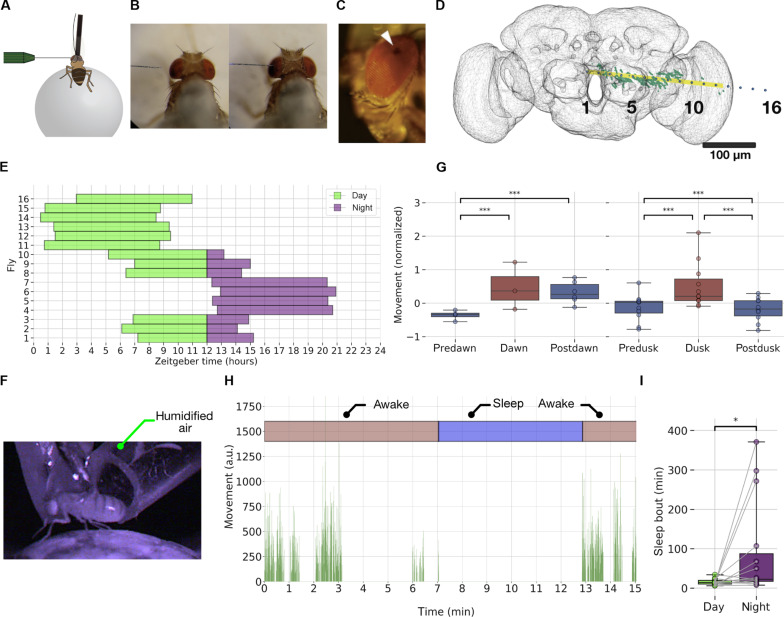
Flies displayed sleep/wake transitions during long-term multichannel recordings with silicon probes. (**A**) Tethered flies were placed on an air supported ball setup that served as a platform for walking/resting. (**B**) Top view of electrode insertion process, with electrode approaching from the left eye of an example fly. (**C**) Side view of electrode insertion site on the dorsal part of the left eye. (**D**) Localization of electrodes using fluorescent dye: The electrode numbers (black) are displayed along with the dye (green) and eigenvector (yellow) indicating the main path of the probe, the FAFB14 neuropil (gray). (**E**) Plot showing the used recording times (LFP) for the 16 flies. Only the first 8 hours of LFP recording was used for analysis though all the flies survived for more than 12 hours. (**F**) Fly movement is quantified using video recorded in profile view with infrared lighting. Humidified air ensures survival. Movement was quantified across adjacent frames using pixel difference and contour thresholding. (**G**) Recorded flies display increased activity (movement) at dawn compared to predawn and dusk compared to pre- and postdusk, thereby showing an expected crepuscular pattern of activity. ***P < 0.001. (**H**) Movement area (in arbitrary units) plotted along with awake and sleep state labeling for an example sleep bout. (**I**) Sleep bouts during the day are significantly longer than during the night. *P < 0.05.

We recorded LFPs across the fly brain using a linear 16-channel electrode inserted into the left eye of flies in a similar context as above, walking (or resting) on an air-supported ball (Fig. 2, A and B). The electrode insertion location was positioned to sample LFPs from the retina to the central brain (Fig. 2C, white arrowhead) (*16*). The depth of insertion of the electrode was optimized using a visual stimulus calibration protocol, based on a reliable LFP polarity reversal identified in the fly inner optic lobes (fig. S2 and see the “Polarity reversal” section). The change in polarity (positive to negative deflections in response to a periodic visual stimulus) was always positioned between electrodes 11 and 13 in all flies, before the start of the long-term LFP recordings. This LFP polarity–based method allowed us to maintain a level of recording consistency across flies in terms of spatial locations of the electrodes, thereby allowing us to compare and combine LFP data across multiple flies. To further ensure reproducible recording locations, we also developed a dye-based registration method (figs. S3 and S4 and see the “Dye-based localization” section) and estimated recording channel locations in the brain for two sample flies. Using this method, we identified three broadly defined brain recording regions to simplify our subsequent analyses (Fig. 2D): central channels (1 to 5), middle channels (6 to 10), and peripheral channels (12 to 16), here grouped by polarity reversal in channel 11. In addition, for further analysis, as the polarity reversal channel is used for re-referencing, the number of channels used in analysis becomes 15.

We used the above calibration steps and recorded LFP data from 16 flies over the course of a day and night cycle (Fig. 2E and see the “Movement analysis” section for data exclusion criteria). We designed our recordings so that experiments were started at different times in different flies to achieve complete coverage of a full day-night cycle. We, however, only examined the first 8 hours of LFP data in each fly (Fig. 2E), to ensure that we were always recording from active and responsive animals (all 16 flies were still alive after 12 hours). The behavior of the flies was recorded under infrared lighting (Fig. 2F), and their movements were quantified using a combination of pixel difference (*12*) and contour thresholding between neighboring frames (see the “Movement analysis” section). As flies are known to be crepuscular in nature (more active in the twilight periods—dawn and dusk), we exploited this activity characteristic to confirm that our subjects were healthy. We analyzed their activity patterns across different crepuscular periods (before and after dawn and dusk periods). For both the dawn and dusk periods, the “crepuscular-type” model (where the movement depends on the crepuscular type; before/after dawn and before/after dusk) emerged as the winning model and a reliable main effect of crepuscular type was found (*P* < 0.01 in dawn and *P* < 0.001 in dusk). Further, we performed post hoc tests using Tukey adjustment (for multiple comparisons) to identify differences between pairs that are significant. We found that movement activity was higher in dawn periods compared to predawn and higher in dusk periods compared to both predusk and postdusk periods (Fig. 2G). For details, refer to the “Crepuscular analysis” section and “Models for movement pattern across crepuscular periods” section under the “Multilevel models” section. This shows that flies remain healthy and active in the recording preparation. To further confirm that the recording preparation is not detrimental, we compared average activity levels across the 8 hours of recording time for each fly (fig. S5A). We found that flies were on average significantly more active the first hour, but then average activity levels remained the same for the following 7 hours. This suggests that after an initial “settling in” period of increased activity, health remained robust for the duration of the recordings that were used in our sleep/wake analyses. For details, refer to the “Models for movement pattern across recorded hours” section.

Sleep was defined by 5-min immobility criteria, based on previous observations in unrestrained flies (*1*, *2*) and tethered flies (*12*, *13*). Fly mobility along with classification of different behavioral states (“awake” and “sleep”) for an example sleep bout is shown in Fig. 2H. Since it was unclear whether flies would even sleep in this multichannel recording preparation, we tallied immobility bout durations across the day and the night for each fly (here, we used 16 hours of video data for each fly all of which survived; see the “Movement analysis” section for data exclusion criteria), expecting that flies should be sleeping more at night on average. We found that flies were able to sleep in this preparation and that nighttime sleep bouts were indeed longer than daytime sleep bouts [median = 22.42 min versus 13.99 min, respectively; *t*(13) = −2.32, *P* < 0.05] (Fig. 2I). This confirms that similar to single-channel LFP recordings (*12*, *13*), flies slept reliably in this multichannel recording preparation, allowing us to assess changes in LFP activity across the fly brain during sleep and wakefulness and to relate these changes to sleep microbehaviors.

Having confirmed that flies are able to sleep in our recording preparation, we next cross-validated the consistency of our LFP recordings across recording hours, to ascertain that LFP quality was not changing as a function of recording duration. We computed the average LFP power spectrum across all channels in the awake and sleep periods across the eight recording hours (hours 1 to 8) for each fly. We found that for both awake and sleep periods, none of the recording hours differed from each other on average (fig. S5, B and C), indicating that an awake or a sleep epoch at the beginning of the recording is quantitatively similar to an awake or sleep epoch at the end of the recording (here, 8th hour) or at other recording hours. This shows that brain activity remains as robust after 8 hours of recording, validating this restricted time frame for our LFP analyses. For details, refer to the “Models for LFP power spectrum across recorded hours” section.

### LFP differences across the brain during spontaneous sleep and awake

**Fig. 3.**
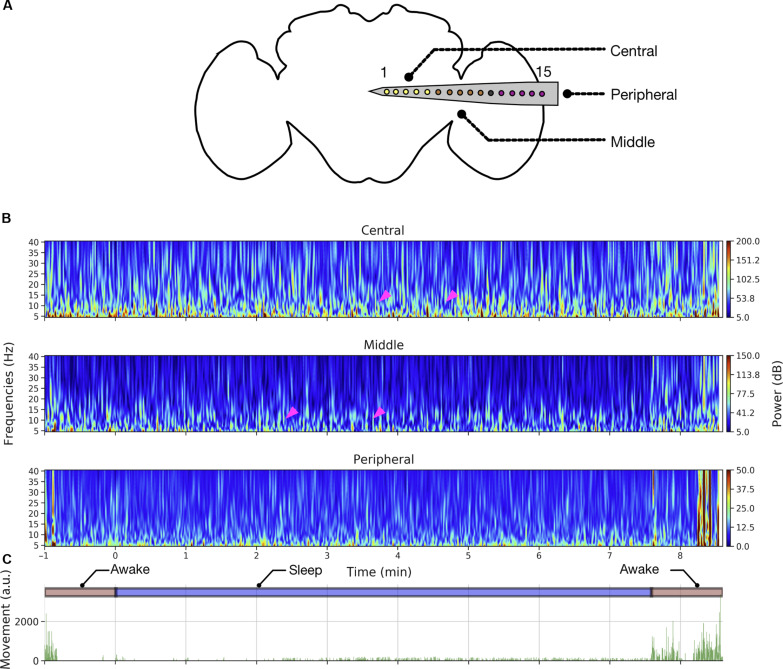
Spectrograms reveal differences across the fly brain during sleep. (**A**) Sixteen electrodes color-coded by location (purple, peripheral; gray, reference; brown, middle; yellow, central) are illustrated on an outline of a standard *Drosophila* brain. (**B**) Spectrogram in different channel groups across an example sleep bout shows variation (magenta arrowheads) in the lower frequency bands (5 to 10 Hz) within the sleep bout, while activity across 5 to 40 Hz in the flanking awake period. (**C**) Movement area (activity pattern) plotted along with awake- and sleep-state labeling for this example sleep bout.

**Fig. 4.**
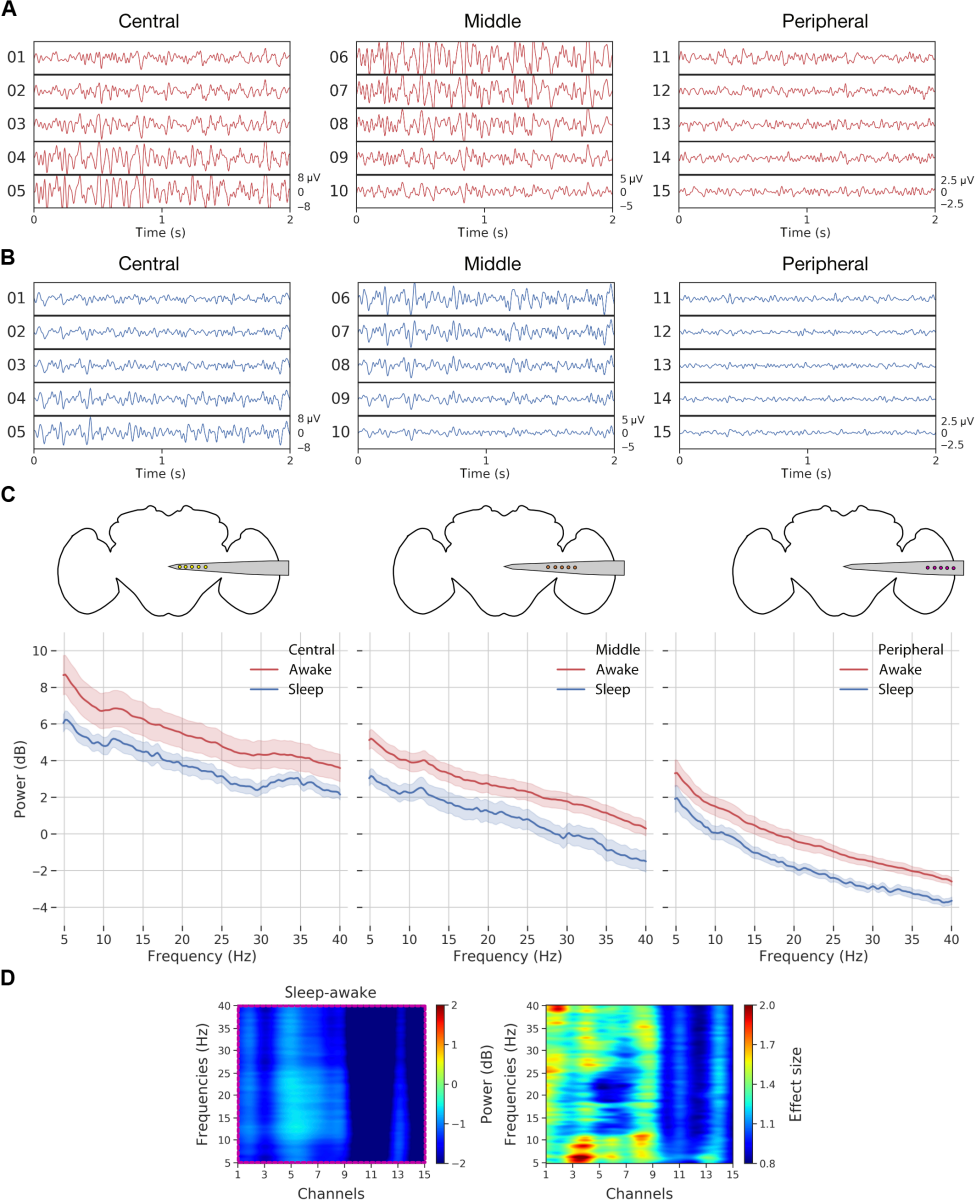
LFP activity decreases proportionally across the entire fly brain during sleep. (**A** and **B**) LFPs across a sample awake and sleep bout in an example fly. (**C**) Mean power spectrum (±SEM) of LFP (5 to 40 Hz) across awake and sleep states in the central, middle, and peripheral channels in an example fly. Across all channels, sleep periods have lower LFP power compared to the awake periods. (**D**) Spectrogram showing the mean difference across sleep and awake periods. Clustering analysis (left) reveals a single significant cluster or ROI (magenta box) across all 15 channels and frequencies from 5 to 40 Hz. Effect sizes (right) are also plotted to identify the individual effect values for every frequency and channel pair.

Next, we focused on the multichannel data to identify potential differences between sleep and wake across the fly brain, separating our recordings into three broad regions: central, middle, and peripheral (Fig. 3A). An example sleep bout and its corresponding spectrograms across the central, middle, and peripheral channels reveal increased activity during sleep in the central brain compared to the periphery (Fig. 3B). In addition, we noted variegated effects in the lower frequencies (5 to 10 Hz) within the sleep bout (Fig. 3B, arrowheads) and significant LFP activity (5 to 40 Hz) associated with locomotion. When we examined sample LFP data more closely across all channels (Fig. 4), we observed higher LFP amplitudes in the central and middle channels than in the peripheral channels and more activity during wake than during sleep (Fig. 4, A and B).

The fly brain is not necessarily quiet during sleep, with some channels (e.g., channels 5 to 7) displaying increased activity compared to other channels (Fig. 4B). To substantiate our observations, we performed spectral analysis on the data. For this purpose, we epoched the LFP data into 60-s bins and computed the power spectrum per epoch per channel [see the “Preprocessing” and “Power spectrum analysis (sleep versus wake)” sections under the “LFP analysis” section]. Since LFP data recorded from flies can be sensitive to physiological artifacts such as heartbeat and body movements (*16*), we used a common referencing system (based on a brain-based signal) that allowed for removal of nonbrain-based physiological noise. Plotting the power spectral density across the three different channel groupings for different frequency bands (5 to 40 Hz) revealed consistently greater power in an example fly during wake than during sleep across the entire recording transect (Fig. 4C). Although decreased LFP power during sleep is consistent with previous findings involving single-channel recordings in flies (*10*–*12*), it was unexpected to see that even the fly optic lobes are significantly less active during sleep compared to wake, suggesting a brain-wide effect.

We next examined more closely the relationship between individual channels and LFP spectral frequency between sleep and wake states. We used nonparametric resampling tools to identify the precise patterns (frequency × channel pairs) differing across awake and sleep at the group level. The outcome of the cluster permutation analysis would be regions of interest (ROI) or clusters across the frequency × channel space that differs between sleep and wake states. For this purpose, we first computed the difference in mean spectral data across wake and sleep for individual flies. Then, we performed a cluster permutation test (flies × frequencies × channels) on the difference between wake and sleep data (Fig. 4D, left) to reveal one significant cluster i.e., ROI (frequency × channel pair) encompassing all frequencies between 5 and 40 Hz and all channels (1 to 15) (Fig. 4D, left). This confirms the spectral results (at group level) in Fig. 4C that showed a brain-wide decrease in power during sleep compared to wake. For details, refer to the “Power spectrum analysis (sleep versus wake)” section. We then sought to identify subclasses of frequencies and channels within this significant cluster that might be more specifically associated with sleep.

To do this, we computed the effect sizes for every channel × frequency combination (Fig. 4D, right). This revealed an interesting frequency structure distinguishing sleep from wake. This included areas of interest in the 5- to 10-Hz and 25- to 40-Hz range in the central channels (channels 1 to 3). A 7- to 10-Hz frequency effect was identified in a previous study as being relevant to sleep transitions in *Drosophila* (*12*), and the higher 25- to 40-Hz range overlaps with frequencies associated with attention-like behavior in flies (*31*, *32*). Consistent with previous work, it is, however, clear that LFP activity is mostly decreased during all of sleep compared to wake, even in the 7- to 10-Hz range that has been associated with sleep transitions (fig. S6).

### LFP differences during induced sleep

Sleep can be acutely induced in *Drosophila* by using optogenetic or thermogenetic activation of sleep-promoting neurons (*33*). We were curious whether induced sleep revealed similar effects across the fly brain, following the same statistical approaches used above for spontaneous sleep. For this, we focused on whole-brain recordings taken from 104y-Gal4/UAS-TrpA1 flies, a sleep-promoting line (fig. S7A) that expresses a temperature sensitive cation channel in the fan-shaped body in the central brain and other regions of the brain (*34*). As shown in a previous study (*12*) and other *Drosophila* sleep studies (*35*), activating these neurons with Transient receptor potential A1 (TrpA1) (by increasing the temperature to ~29°C) results in behavioral quiescence and induced sleep, whereas control strains remain awake and active. In these recordings, a different multichannel probe was used (fig. S7B), with 16 recording sites that spanned the entire brain from eye to eye (*16*). We preprocessed the induced sleep LFP data (see the “Thermogenetic sleep induction” section) in a similar fashion to our spontaneous sleep LFP data. We first contrasted the mean power spectra per fly under two conditions: baseline and sleep induction (fig. S7C). As above, we then performed a cluster permutation test (flies × frequencies × channels) on the difference between baseline wakefulness and induced sleep to reveal a significant cluster (frequency × channel pair). Thus, we uncovered a significant cluster (fig. S7D) in the central brain channels across all (5 to 40 Hz) frequency bands, whereas the 104y-Gal4/+ control flies did not reveal such a cluster (fig. S7, E and F). Note that sleep induction using this strain yielded an opposite effect to what we found during spontaneous sleep: LFP activity during induced sleep is on average higher than during baseline wakefulness (fig. S7D), while it was lower during spontaneous sleep (Fig. 4D). In addition, the effect observed during induced sleep was only observed in the central channels, whereas the spontaneous sleep effects appear to at least cover the entire hemisphere from center to periphery. This shows that genetically induced sleep in flies can produce notably different electrophysiological signatures than spontaneous sleep, consistent with several previous similar observations (*12*, *17*, *36*–*38*). For the rest of this current study, we focus on spontaneous sleep.

### Distinct sleep stages identified by machine learning

**Fig. 5.**
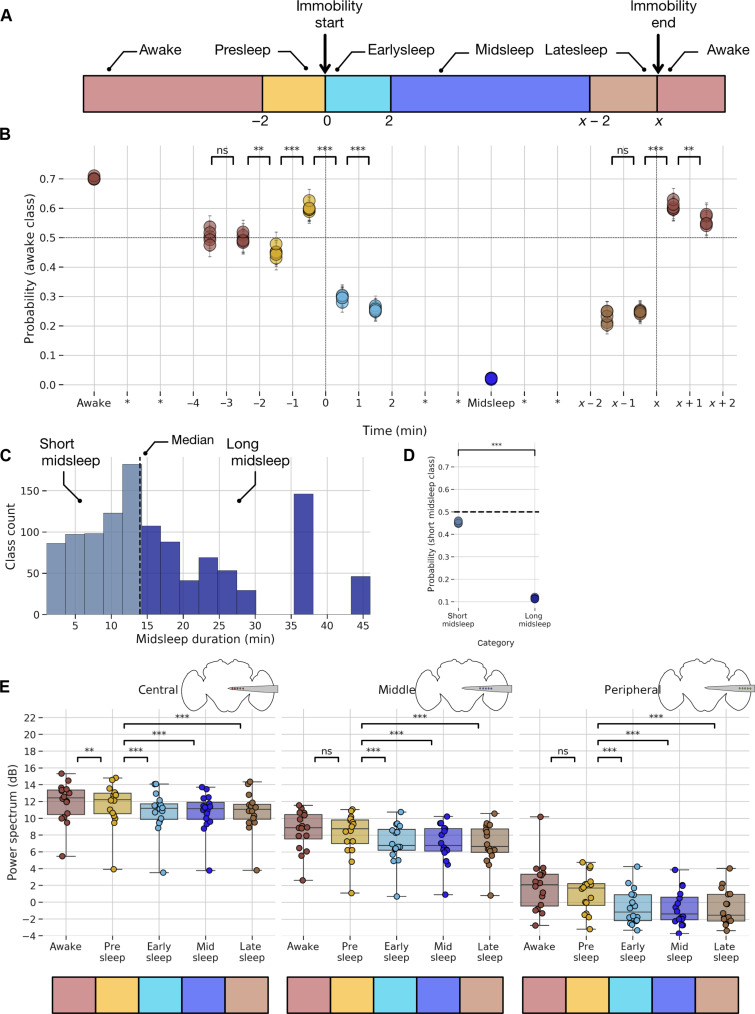
Machine learning reveals distinct sleep stages in the fly brain. (**A**) Sleep bouts (>5 min) were binned into four segments. Two minutes before the start of immobility (presleep), 2 min after the start of immobility (earlysleep), and 2 min before the end of immobility (latesleep), the period between early and latesleep is midsleep, and rest of the periods is categorized as awake. (**B**) Probability estimates (of awake class) plotted across different time segments. Horizontal dashed line indicates that values above 0.5 are likely to be classified as awake and below 0.5 as sleep. Vertical dashed line at 0 indicates start of immobility period, and that at *x* indicates end of immobility period. Unpaired sample *t* test was conducted across different epochs to test for statistical significance. (**C**) Histogram indicating the different durations of the classes of the midsleep epochs. Vertical dashed line indicates median value below which epochs are considered short midsleep and above which epochs are considered as long midsleep. (**D**) Probability estimates (of short midsleep class) of a classifier trained to differentiate short versus long midsleep class plotted across categories. Horizontal dashed line indicates that values above 0.5 are likely to be classified as short midsleep and values below 0.5 are to be classified as long midsleep. Unpaired sample *t* test was conducted to test for statistical significance. ***P < 0.001. (**E**) Comparison of mean power spectrum across different channels and different sleep stages. **P < 0.01 and ***P < 0.001.

Our earlier analysis of microbehaviors during sleep in this preparation (Fig. 1) suggests that sleep is not a single phenomenon and that the requisite 5-min immobility criterion might not fully capture potential LFP and behavioral changes that could occur across a sleep bout. There is evidence that sleep quality (via arousal threshold probing) in wild-type *Drosophila* flies also changes across a bout of quiescence (*13*, *39*), suggesting that flies transition from lighter to deeper sleep stages. To assess whether this might also be evident in our multichannel recordings, we divided our LFP data (for all channels) into five different temporal segments, analyzing only sleep epochs that were 5 min or longer (Fig. 5A): (i) “presleep”: the 2 min (−2 to 0 min) before flies stopped moving; (ii) “earlysleep”: the first 2 min (0 to 2 min) after the start of a sleep bout; (iii) “latesleep”: the last 2 min of sleep before mobility resumed; (iv) midsleep: any time between earlysleep and latesleep; and (v) awake: the rest of our LFP data. Our partitioning of the LFP data matches a similar partitioning applied to whole-brain calcium imaging of flies engaged in spontaneous sleep (*17*).

To examine how LFP-based signatures change within a sleep bout, we decided to perform a hypothesis-agnostic analysis through machine learning techniques. To perform this machine learning–based classification, we first used SVM-based techniques. Briefly, SVM belongs to a class of supervised learning model, which is composed of building a hyperplane or set of hyperplanes in a high-dimensional space (using the kernel trick for nonlinear mapping functions) with the goal to maximize the separation distance between the closest data point (in the training dataset) of any class (functional margin) (*40*). The choice of the optimal hyperplane is made in such a way that the generalization error would be lower for the new data points in the test dataset (fig. S8A). For detailed steps for preprocessing of data and implementation of classifiers, refer to the “Sleep staging by classifiers” section. The probabilistic prediction per class per iteration is shown in Fig. 5B. It is interesting to note several points. First, the probability of awake data is ~0.7, and that of midsleep is ~0.0, indicating that the classifier performs well on classes that it has already been trained on. Second, at the epoch −2 to −1 min, when the fly is still moving (yellow circles), LFP data indicate that it is closer to resembling sleep (<0.5), before dropping fast to ~0.3 (turquoise circles) in the first 2 min of sleep.

The above analysis indicates that with this approach, we could predict the probability that a fly will fall asleep 2 min before the start of the immobility period. Just 1 min before flies fall asleep, the LFP data indicate a brief moment more closely resembling wake (yellow circles), perhaps associated with grooming periods [observed in honeybees, for example (*41*)]. The first 2 min of sleep (turquoise circles) reveals a probability metric halfway between midsleep and wake, suggesting either a gradual descent into deeper sleep or a distinct sleep stage. Last, at the epoch from *x* − 2 to *x* − 1 min before mobility resumes (brown circles), the probability metric returns to a similar level as early sleep. Immediately after mobility resumes, the LFP data are classified as no different from awake, i.e., there is no postsleep ambiguity. Note that only the awake and midsleep data have been seen by the classifier, the rest of the data −4 to +2 min and *x* − 2 to *x* + 2 min have never been seen by the classifier. In addition, midsleep collapses a wide range of different sleep durations in different flies, so it could still be averaging different sleep states within. Nevertheless, our results suggest that broadly dichotomizing midsleep and wake identifies other sleep (and wake) stages that resemble neither.

To confirm this, we next examined whether midsleep episodes of different durations are different from each other. We first plotted the different durations of classes of the midsleep episodes (Fig. 5C). On the basis of a distribution centered around a median, we defined midsleep episodes of <14 min as short midsleep and >14 min as long midsleep. We next used an SVM-based classifier (as before) but trained to distinguish between short and long midsleeps. We identified the probability estimates of the short midsleep class (Fig. 5D) on both the short and long midsleep categories. If short and long midsleeps are different from each other, then they should follow two characteristics, similar to those established by the classifier trained on awake versus midsleep. They are the following: (i) The awake class displayed probability values ~0.7 and midsleep around ~0.0, so the values of the trained classes were as different from each other and different from 0.5 (chance) as well, indicating that the classifier has identified features able to differentiate between awake and midsleep classes. (ii) The awake and midsleep class probability values differed significantly from each other (indicating stability of values) across different classifier train/test iterations. When these two criteria were applied to the case of classifier trained on short midsleep versus long midsleep, they satisfy the latter criteria (significantly different and thus stable classifier performance) but not the former criteria as short midsleep values of ~0.4 and long midsleep around ~0.0, and this suggests that the classifier did not find features that are clearly able to differentiate short and long midsleep classes and, hence, different midsleep durations display similar LFP qualities across the fly brain. Whether these include intercalated epochs of different quality sleep remains an open question.

### Model-based spectral analysis across different channels

Having revealed how multichannel LFP data can be used to differentiate across different temporal stages of sleep, we next decided to identify what channels might be important for revealing this. For this purpose, we used a multilevel modeling approach. To reveal how spectral data might change throughout the fly brain across a sleep bout, we calculated the mean spectral power for each of the aforementioned epochs and pooled data from central, middle, and peripheral channels. Because different flies had varying numbers of sleep epochs, we used multilevel models instead of traditional repeated measures of ANOVA. For details, refer to the “Models for spectral analysis” section. To understand the modulation of the LFP power spectrum by sleep epoch, we defined multiple models: a null model, where the power spectrum depends on the mean per fly; an epoch model, where power spectrum depends on the LFP epoch type (wake or sleep); a channel model, where power spectrum depends on the LFP channel (central, middle, or peripheral); an epoch channel model, where power spectrum depends on a combination of epoch type and LFP channel type. The “epoch channel” model emerged as the winning model. In the epoch channel model, we found that there was a reliable main effect of both epoch (*P* < 0.001) and channel (*P* < 0.001) on power spectrum and the interaction between epoch and channel also had a reliable effect (*P* < 0.001) on power spectrum. In summary, the above model-based analysis confirms that the power spectrum of the LFP data varies on the basis of the channel location and the epoch state of the fly.

We then proceeded to examine more closely how differences in the sleep LFP might be segregated across the fly brain (Fig. 5E) using post hoc tests (using Tukey adjustment for multiple comparisons) from the epoch channel model. In the central channels, the awake data were significantly different compared to all sleep categories and critically were also different to the presleep data. Note that, behaviorally, the fly is still considered awake in the presleep period (i.e., it is still moving). Thus, the ability to predict sleep at least 2 min before the onset of immobility, which was revealed in our SVM analysis (Fig. 5B), might be explained by these significant spectral differences only observed in the central channels. In the middle channels, the awake data were also significantly different across all sleep categories but was not different to the presleep data. Further, the presleep period was significantly different from earlysleep, midsleep, and latesleep periods. In the peripheral channels, the awake data were significantly different across all sleep categories but were again not different to the presleep data. Together, mean power spectral data across different channels were thus able to differentiate between awake, presleep, and different sleep epochs of sleep. However, the post hoc analysis did not differentiate among sleep epochs (earlysleep, midsleep, and latesleep). Since this is inconsistent with previous findings using single glass electrodes (*12*), we questioned whether the pooling of channel × frequency data (three broad brain regions × one overall power spectrum) could be hiding more specific effects that might become evident with the full (15 × 145) dimension of channels × frequencies.

### LFP features across different temporal stages of sleep

**Fig. 6.**
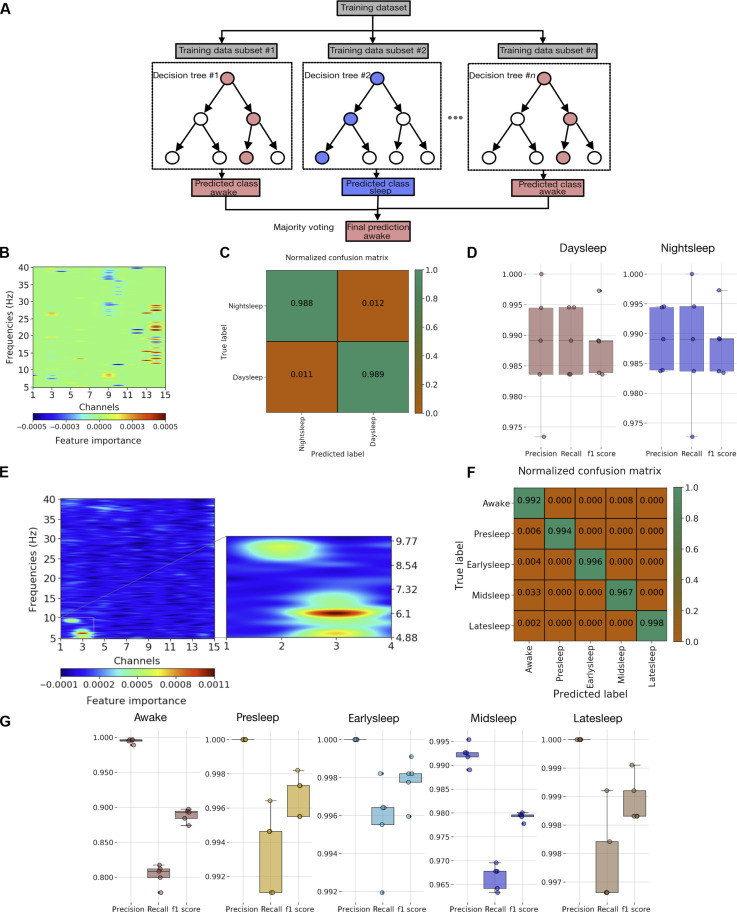
Classifiers identify specific spatial and frequency domains in the fly brain. (**A**) Schematic indicating the workings of a multiclass random forest classifier in identifying the predicted class. Random forest classifier was trained to differentiate across sleep during day and night periods. (**B**) Feature importance reveals an ROI across peripheral channels and frequency bands (10 to 30 Hz) as critically important features. (**C**) Normalized confusion matrix. (**D**) Performance metrics: precision, recall, and F1 score. Multiclass random forest classifier was trained to differentiate across awake and different sleep states. (**E**) Feature importance reveals an ROI across central channels and frequency bands (5 to 10 Hz) as critically important features. (**F**) Normalized confusion matrix. (**G**) Performance metrics: precision, recall, and F1 score per class.

Having established the existence of different temporal stages of sleep using a classifier based on SVM and confirming the same using model-based analysis, we were next interested in the features in the LFP data (which channels at what frequencies are important for distinguishing epochs within a sleep bout) that help us differentiate these stages. For this purpose, we used random forest classifiers. A random forest classifier is a class of supervised learning algorithms that uses an ensemble of multiple decision trees for classification/regression. This could be illustrated by an example (Fig. 6A). In the first step, subsets of training data (#1 to #*n*) were created by making a random sample of size *N* with replacement. This allows for the ensemble of decision trees (#1 to #*n*) to be decorrelated, and the process of this random sampling is called bagging (bootstrap aggregation). In the second step, each decision tree (#1 to #*n*) picks only a random subsample of features (feature randomness) instead of all features (again allowing for the decision trees to be decorrelated). In the final step, all the decision trees create individual predictions of classes, and the final outcome would be resolved by simple majority voting (illustrated here with a goal of classifying awake versus sleep). Thus, bagging and feature randomness allow for the random forest to perform better than individual decision trees. Furthermore, we also computed classifier performance metrics (see the “Classifier metrics” section) such as precision, recall, F1 score, and normalized confusion matrix for evaluation. We also used a permutation importance technique (see the “Multiclass random forest classifier analysis and feature importance” section) to identify the relative importance of features in the performance of classifiers, thereby identifying physiological features (channels × frequency) that are important for differentiating across categories.

We first decided to use the random forest classifiers to determine whether there were any differences between day and night sleep, hence performing classification across the classes: “daysleep” and “nightsleep.” We identified LFP features (Fig. 6B) discriminating across daysleep and nightsleep in the peripheral channels across frequency bands (10 to 30 Hz), consistent with a previous study using single-channel LFP (*13*). We also computed the normalized confusion matrix (Fig. 6C), which revealed excellent performance in predicting the daysleep and nightsleep classes. Classifier performance metrics across the daysleep and nightsleep classes shown in (Fig. 6D) also indicate good performance across classes (>0.9).

We then performed a multiclass classification of the following classes: awake, presleep, earlysleep, midsleep, latesleep, and identified important LFP features (Fig. 6E) discriminating across categories. The most important features fall within a narrow range of channels (1 to 3) and frequencies (5 to 10 Hz). This indicates that the 5- to 10-Hz frequency range within the central channels is the most important in resolving different sleep stages. We also computed the normalized confusion matrix (Fig. 6F), which revealed excellent performance in predicting the multiple classes (green boxes). This indicates that classifier features (channels × frequency) are sufficient to distinguish multiple sleep stages (classes) and furthermore provide direct evidence of multiple sleep stages with distinct frequency components. Classifier performance metrics across the target classes shown in (Fig. 6G) also indicate good performance across classes for the different sleep segments (>0.9).

Last, we also cross-validated the utility of the permutation-based technique in identifying important features across epochs. For this purpose, we created a multiclass random forest classifier, with target classes as awake, sleep, and identified the features that are important in this classifier (fig. S9A). The most important features are actually distributed evenly among all the features (channels × frequency), thus cross-validating our previous clustering results (Fig. 4D, left), wherein we showed that the LFP differences across awake and sleep are distributed across all channels and frequencies.

### PE behavior during sleep in multichannel recordings

**Fig. 7.**
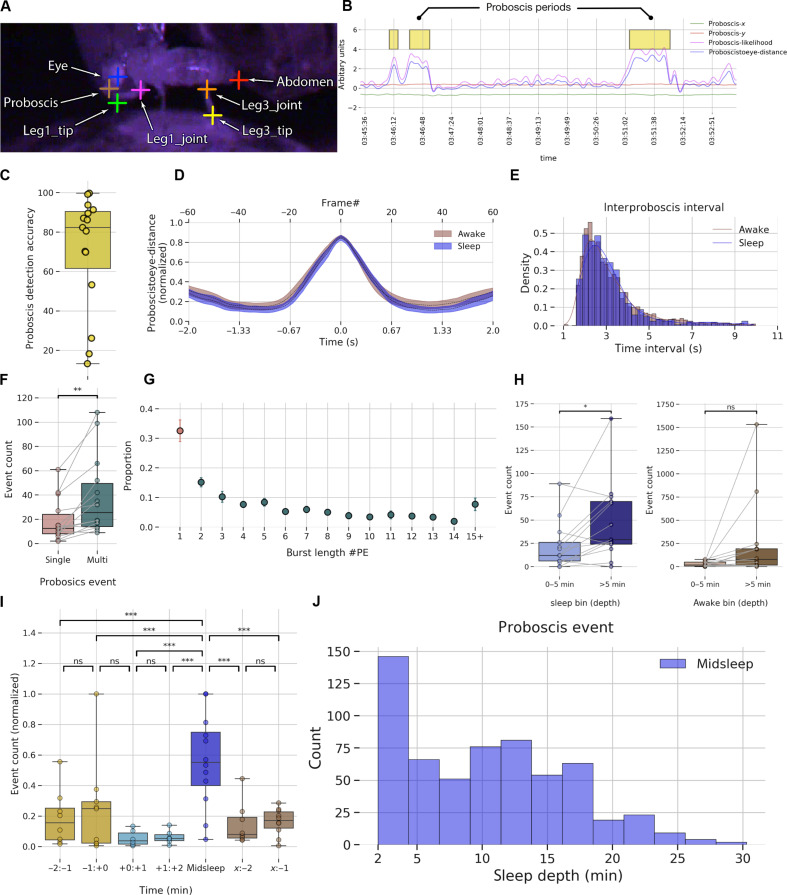
Recorded flies still display microbehaviors characteristic of deep sleep. (**A**) Seven different body parts were annotated using DeepLabCut for pose estimation. (**B**) Identified PE periods (yellow boxes) were plotted along with filtered body parts of proboscis and other estimated metrics. (**C**) PE events detection accuracy across different flies. (**D**) Average proboscis to eye distance (across all flies) plotted across frames and time periods is similar in awake and sleep states. (**E**) During PE burst events, interproboscis intervals are highly regular, with one PE occurring every 1.5 s at group level. (**F**) PE events are more likely to occur as multiple events (bursts) instead of a single event. (**G**) About 33% of PE events occur as single, while the rest are bursts of varying length. (**H**) Number of PE events occurring after the first 5 min of sleep is significantly higher than in the first 5 min, indicating that more PE events occur in deeper stages of sleep. Also displayed is the control analysis, with awake depth showing no increase with PE event count. (**I**) Normalized proboscis event count across different sleep segments: −2:−1 indicates 2 min before start of sleep, −1:0 indicates 1 min before start of sleep, +0:+1 indicates 1 min after start of sleep, +1:+2 indicates 2 min after start of sleep, *x*:−2 indicates 2 min before end of sleep, and *x*:−1 indicates 1 min before end of sleep. The normalized count is significantly higher in the midsleep segments compared to other segments. (**J**) Proboscis events occurring in midsleep across different sleep depths. *P < 0.05, ** *P* < 0.01, and ***P < 0.001.

Earlier, we identified that rhythmic PEs during midsleep (Fig. 1), which we propose, describe a distinct sleep stage in *Drosophila* (*27*). However, it is unclear whether brain activity associated with PEs is sleep-like or PE-specific. This distinction is important, as it would disambiguate a unique brain state (deep sleep) from a specific behavior associated with that state (PEs). To identify PEs in our electrophysiological dataset, we again used DeepLabCut (*30*) to track different body parts of the fly (Fig. 7A). We further used multiple classifiers based on the tracking data, followed by manual verification to identify the PEs. Sample PEs in an example fly along with a few of the features (*x*,*y* proboscis location, likelihood of location, and distance of proboscis to eye) are shown in Fig. 7B. For more details on the proboscis detection steps, refer to the “Proboscis tracking for flies on electrophysiology setup” section. Our classifier accuracy was over 80% for most flies (Fig. 7C): The ground truth was validation by a human observer on classifier detected events. In Fig. 7D, we plot the mean proboscis to eye distance for all the flies averaged across awake and sleep bouts. As described earlier for flies without implanted electrodes, PEs executed during wake and sleep are behaviorally similar and, hence, would be difficult to distinguish from each other using video alone. Similar to our behavioral dataset, PE events usually occur in rhythmic bouts, rather than single events. In Fig. 7E, we plot the interproboscis interval period, which is the interval between consecutive PE events in a single proboscis bout. Most proboscis events occur within 1.8 s (95th percentile) of each other. As shown before in our behavioral data without implanted electrodes, the interproboscis interval does not vary across awake and sleep periods. Next in Fig. 7F, we decided to probe the number of single (one PE event) and multiple (>1 PE event) across different flies. We found that occurrences of single PE events (both across wake and sleep periods) are significantly lower than multiple PE events using a pairwise *t* test with *t*(13) = 3.72, *P* < 0.01. To further illustrate this point in Fig. 7G, we plotted the burst length of a PE event (number of extension events within a PE bout) and found that only 33% of the events are single PE, while the rest are multiple PE events. Overall, our investigation of PEs in this multichannel recording dataset is in concurrence with our first (electrode-free) dataset, suggesting that inserting probe into the fly brain does not alter several measures associated with this microbehavior.

Previous work has linked PEs with a deep sleep stage in flies (*27*). We therefore next investigated whether the number of PEs varied across a sleep bout in our LFP recording dataset, as suggested in our purely behavioral dataset (Fig. 1G). We found that more PE events occur after 5 min of a sleep bout, compared to those occurring before the 5th min of sleep (Fig. 7H) [pairwise *t* test, *t*(12) = −2.8, *P* < 0.05], suggesting that PEs indeed predominate during later stages of sleep. We also compared PEs immediately after flies had awakened from sleep, which revealed no significant difference (Fig. 7H) [pairwise *t* test, *t*(13) = −1.92, *P* > 0.05] between PE bouts occurring after the 5th min of an awake bout compared to those occurring before the 5th min of an awake bout, confirming that transitions into sleep (rather than transitions back to wake) were associated with increased PE events.

We next asked whether the number of PE events changed across a sleep bout in our multichannel recording preparation. To determine whether the PE event count varies across different temporal sleep stages (Fig. 7I), we used multilevel models. For details, refer to the “Models for PE event counts” section. The time_label model (where the PE event count depends only on the specific temporal sleep stage) emerged as the winning model. Further, we performed post hoc tests using Tukey adjustment (for multiple comparisons) to identify differences between pairs that are significant. We found that PE events occur more often in midsleep compared to other sleep stages. Returning to our original observation that most PEs occur after 5 min of sleep, we plotted the distribution of PE events occur in the midsleep epoch across all flies (Fig. 7J) and found that 95 percentile of all PE events in midsleep indeed occur after 2.5 min of the midsleep epoch (thus, 4.5 min from sleep onset).

### LFP features of a deep sleep stage with PEs

We next questioned whether PEs occurring during sleep and wake had similar neural correlates or whether the sleep-related events were indeed different and thus indicative of a unique sleep-related function. We therefore focused on the multichannel data to identify any differences in the LFP activity associated with PEs during wake and sleep epochs. We first identified the PE periods (refer to the “Identification of proboscis periods” section), extracted the LFP data, and epoched them into 1-s bins. Second, we used spectral analysis to determine whether epochs characterized by PEs differ in frequencies across different channels for wake compared to sleep. For this purpose, we computed the spectral power for every 1-s epoch per channel (see the “Power spectrum analysis” section), using as before a common reference system for re-referencing the LFP data. Third, we used nonparametric resampling tools to identify the precise patterns (frequency × channel pairs) differing in proboscis periods within awake and sleep at the group level. For this purpose, we first computed the difference in mean spectral data across nonproboscis periods (awake or sleep) and proboscis periods (awake proboscis and sleep proboscis, respectively) for individual flies. We then performed a cluster permutation test (flies × frequencies × channels) on the difference data to reveal significant ROIs or clusters (frequency × channel pair).

**Fig. 8.**
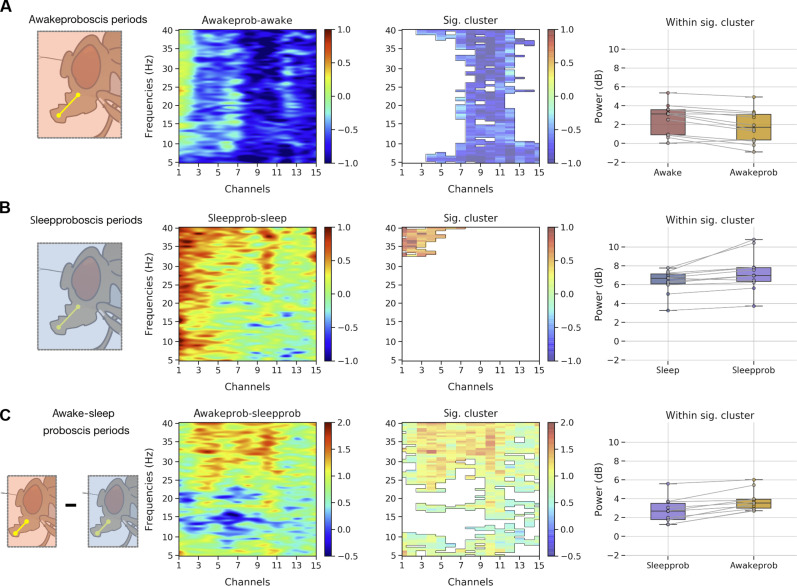
Sleep-related PEs have distinct neural correlates. (**A**) Spectrogram showing the mean difference across awakeprob (PE events in awake periods) and awake periods, while clustering analysis reveals a single significant cluster across middle channels in all frequencies. Activity within the significant cluster indicates that activity in the awakeprob is comparatively lower than awake periods. (**B**) Spectrogram showing the mean difference across sleepprob (PE events in sleep periods) and sleep periods, while clustering analysis reveals a single significant cluster across central channels in higher frequencies (32 to 40 Hz). Activity within the significant cluster indicates that activity in the sleepprob is comparatively higher than sleep periods. (**C**) Spectrogram showing the mean difference across awakeprob and sleepprob periods, while clustering analysis reveals a single significant cluster mostly across all channels in higher frequencies (25 to 40 Hz). Activity within the significant cluster indicates that activity in the sleepprob is comparatively lower than awakeprob periods, thereby elucidating a significant difference across proboscis events occurring in sleep and awake periods (although, phenotypically, they look the same; Fig. 7D).

In Fig. 8A, we show the difference data for awake PE events (awake proboscis–awake period) and clustering analysis, which reveals a significant cluster in the middle channels (channels 6 to 10) across all frequencies. Further, within the significant cluster, we also performed a post hoc analysis, revealing that spectral activity within the awake proboscis periods is lower than awake periods. In Fig. 8B, we show the difference data for sleep PE events (sleep proboscis–sleep period), and clustering analysis reveals a significant cluster in the central channels (1 to 5) across higher frequencies (32 to 40 Hz). Further, within the significant cluster, we also performed a post hoc analysis, revealing that spectral activity within the sleep proboscis periods is higher than sleep periods (in contrast to the awake proboscis periods). In Fig. 8C, we directly compared the awake and sleep proboscis periods and showed the difference data (awake proboscis–sleep proboscis) and clustering analysis, which reveals a significant cluster in the central and middle channels (channels 1 to 9) across higher frequencies (25 to 40 Hz). Further, within the significant cluster, we also performed a post hoc analysis, revealing that spectral activity within the sleep proboscis periods is lower than awake proboscis periods. This suggests that PEs occurring during sleep are qualitatively different from identical PE events occurring during wake. This suggests that the brain activity state [e.g., quiet or deep sleep (*17*, *36*)] overrides the neural correlates associated with the same behavior occurring during wake.

## DISCUSSION

**Fig. 9.**
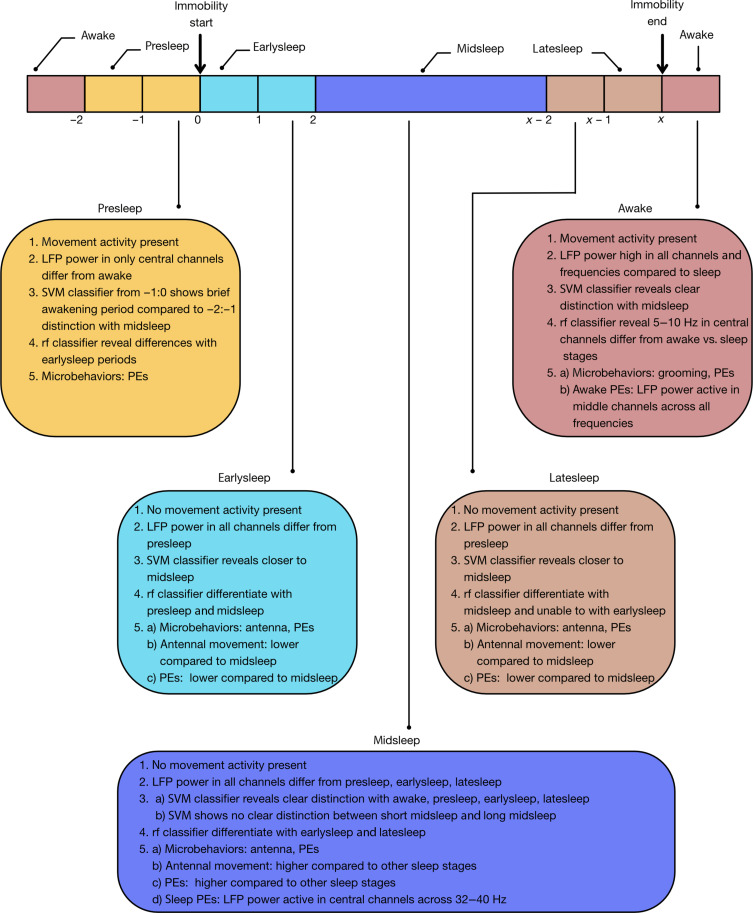
Flies sleep in distinct stages. Schematic summarizing brain and behavior parameters across wake and sleep stages. rf, random forest.

In this study, we used a combination of multichannel electrophysiology, behavior, and machine learning to identify and characterize spontaneous sleep in *Drosophila* flies. We describe distinct features associated with sleep stages in wild-type flies (Fig. 9). However, we expect that mutant animals could reveal different brain or microbehavior dynamics, especially if sleep functions are impaired. Our multichannel recording preparation should allow LFP activity of mutant strains to be characterized and compared to wild-type controls to provide an additional level of explanation than behavioral activity readouts. We have recently published a detailed protocol for performing multichannel recording experiments, which should make this approach more widely accessible (*42*). This approach provides an alternative to optical imaging techniques (*17*, *43*) for assessing whole-brain states. However, for understanding and probing the exact spatial and cellular nature of specific sleep stages identified in this study with higher resolution, innovations in optical imaging during sleep would be required. For example, closed loop techniques could be used to image sleep only during specific stages, such as only during midsleep PE bouts. With the advent of new genetically encoded calcium indicators with faster kinetics and higher sensitivity [such jGCaMP8 (*44*)], it should also be possible to combine genetically encoded calcium indicators and LFPs recorded together to provide a complementary readouts to better understand sleep physiology and functions in this model. Similarly, genetically encoded voltage indicators such as ArcLight (*45*) should reveal whether our findings generalize to other methods of describing electrical activity in the sleeping fly brain.

Sleep is most likely a whole-brain phenomenon, meaning that its presumed varied functions (*46*) are understood to be of benefit to the entire brain rather than to only specific subcircuits. There is good evidence for this in the *Drosophila* model, with synaptic physiology for example changing during sleep in the optic lobes of flies (*47*) and brain-wide (*48*). Similarly, in mammals, subcortical and cortical brain regions experience sleep-related changes that are thought to be important for maintaining neuronal homeostasis (*11*). Accordingly, to better understand sleep in an animal model such as *D. melanogaster* requires sampling associated changes not only in neural activity across the fly brain but also in specific subcircuits of interest. Unlike in larger animal models such as rodents, recording from multiple brain regions in behaving (and sleeping) flies has been challenging, so there has been limited capacity to investigate dynamic brain processes during sleep in this otherwise powerful model system. While genetically encoded reporters of neural activity (e.g., calcium indicators such as GCaMP) have been successfully used to describe spontaneous sleep in flies (*17*, *18*, *49*), these are typically still limited to a narrow ROI (e.g., the mushroom bodies or the central complex), and imaging conditions are rarely commensurate with the typical day-night cycles of normal sleep. In this study, we overcame these drawbacks by recording electrical activity from 16 channels across the fly brain, in behaving flies across long-lasting recordings that spanned a typical day and night. Our multichannel recording preparation therefore approximates as closely as possible—in flies—a sleep EEG, which has been the starting point for most discussions on sleep physiology in other animals. The human sleep EEG has defined the sleep stages that are now being investigated in other animals (*46*, *50*–*52*), although this is obviously a neocortical view with potentially little relevance to animals lacking the neural architecture giving rise to sleep signatures such as delta (1 to 4 Hz) during slow-wave sleep or theta (5 to 8 Hz) during REM sleep (*53*).

Rather than focusing on specific frequency bands such as delta and theta, we conducted an agnostic analysis of our multichannel LFP data using machine learning techniques. These unbiased classifiers identified distinct stages of sleep, in flies that were otherwise entirely quiescent (apart from certain microbehaviors, which we discuss further below). These identified sleep stages align closely with similar changes in brain activity dynamics observed in calcium imaging data in spontaneously sleeping flies (*17*). For example, in the calcium imaging data, we showed that even before sleep onset, the number of “active” neurons is already different (lower) than wake; accordingly, in the current electrophysiological data, the classifiers predict sleep onset 2 min before flies stop moving. This also aligns with an older (single channel) electrophysiological sleep study in flies showing that brain LFP activity becomes uncorrelated from behavior 5 min before sleep onset (*15*). Together, these findings make a compelling case for dissociative states in the fly brain, which is consistent with the view that these states might also be changing within a sleep bout.

Our multichannel recordings also revealed that changes in sleep physiology are likely to encompass the entire fly brain, from the optic lobes to the central complex. This is consistent with a recent study where we found that experimentally induced “quiet” and active sleep engaged different whole-brain transcriptional programs (*36*). That the whole insect brain “sleeps” is also consistent with other studies, although this has not been previously demonstrated using a comprehensive multichannel approach. An early study in honeybees showed that visually responsive neurons in the optic lobes become unresponsive during sleep (*54*) and that these cells become rapidly responsive again when bees are woken up with an air puff. Immunochemical studies investigating synaptic proteins found that these were down-regulated in the optic lobes during sleep (*47*) and in the whole brain (*48*). It is understood that the insect optic lobes receive significant feedback from the central brain and from the contralateral lobes (*55*, *56*), and it has been shown that oscillatory neural activity extends throughout the fly brain (*16*), so our finding that the optic lobes also sleep is expected. Recent work using a similar multichannel recording preparation found that isoflurane anesthesia affected feedback from the central brain to the optic lobes (*24*), suggesting that this efferent communication is a feature of the waking fly brain. However, sleep in the central fly brain is different from sleep in the periphery. Only central channels were predictive of sleep onset, and only the central channels revealed the 5- to 10-Hz frequency features that we have previously identified in single-channel recordings (*12*). Although this hints at a sleep-regulatory role for the central complex, aligning with previous studies (*17*, *34*, *38*), it is important to perform causal experiments involving central complex neurons to clarify the same.

Sleep in *Drosophila* was originally defined by inactivity criteria based on locomotion-based readouts (*1*, *2*). Subsequent studies using video monitoring and probing arousal thresholds confirmed these simple readouts to be accurate estimates of sleep in flies (*13*, *39*, *57*), but these behavioral studies also showed that flies slept in distinct stages. Only recently has closer video monitoring of fly microbehaviors revealed that these animals are not entirely immobile during sleep (*27*), although some microbehaviors were already anecdotally observed in the first reports of fly sleep, such as changes in posture (*1*, *2*). Other insects, such as honeybees, display characteristic microbehaviors during sleep, such as changes in posture (*41*) and antennal movements (*58*). In our study, we also found evidence of altered antennal movements during fly sleep, alongside the previously reported PEs (*27*). These microbehaviors are not necessarily correlated, although they do seem to be increased during mid-sleep epochs. PEs have been associated with a deep sleep function (waste clearance) in a previous study (*27*), so their occurrence in rhythmic spells during mid-sleep is consistent with that interpretation.

PEs during wake and sleep are electrophysiologically different, although they are behaviorally identical. We found that the neural signatures of PEs occurring during wake are concentrated in the middle channels and spread across a broad frequency range (5 to 40 Hz). Note that these middle channels could coincide with the location of neuropils of the antennal mechanosensory and motor center. Several studies (*59*, *60*) have implicated the antennal mechanosensory and motor center as the location of axons of gustatory projection neurons and, thus, an immediate higher-order processing center for taste. Another study (*61*) has also shown that persistent depolarization of motor command activity of the Fdg (feeding) neurons could also result in PEs. In this context, note that LFP activity during PE events in the awake periods is higher than those in the awake periods without PE events, suggesting a distinct PE signature. However, this is not the case for the exact same behaviors during sleep. We found that LFP activity for PEs occurring during sleep bouts is concentrated instead in the central channels and primarily engages the higher frequencies (32 to 40 Hz). This suggests a distinct control mechanism for PEs occurring during sleep versus wake.

There are obviously several drawbacks to studying sleep physiology in a tethered animal that has been skewered by a recording electrode. Sleep cannot be quite normal in such a preparation. For example, it is possible that the damage caused by the electrode evokes an increased need for repair (*62*) and consequently waste clearance (*27*), thus increased PE behavior. However, this would also be the case for windows in the brain created for calcium imaging (*17*) (and in the latter scenario, the proboscis is typically glued in place to prevent brain motion artifacts), so no fly brain recording preparation (yet) can realistically sidestep these concerns. Nevertheless, it is evident that even in this somewhat contrived context, flies do still sleep and their behavior is comparable to tethered preparations without electrodes inserted. In addition, by restricting our analyses to only 8 hours of recording per fly, we ensured that all of our sleep data were generated from healthy animals that were still active when awake. We did not conduct arousal threshold experiments in this study to determine sleep quality, as these experiments inevitably alter sleep architecture, and our main goal was to examine spontaneous sleep in this preparation. Future studies using this paradigm will show how brain responsiveness to stimuli changes across sleep and wake.

In our study, we contrasted our spontaneous sleep analyses with an induced sleep dataset, using a 104y-Gal4/UAS-TrpA1 line [collected as a part of the study (*12*)]. While it is understood that this sleep-promoting line expresses broadly, beyond just the fan-shaped body alone (*63*), it nevertheless still renders flies quiescent and achieves sleep functions (*17*, *38*). Still, sleep-like effects associated with activating this circuit should be interpreted with caution, as it is clear from our data here that although the central brain is activated during this state, it could be the result of neurons other than in the fan-shaped body being targeted as well (*64*). So, whether the observed changes in LFPs in these flies are related to the behavioral sleep effects remains unclear. We do not interpret the increased LFP activity as a seizure-like effect as in (*63*) but rather as a form of active sleep also seen during optogenetic or thermogenetic activation of other sleep-promoting lines (*17*, *35*, *65*). One important observation from our multichannel study, however, is that we never saw a level of increased LFP activity during spontaneous sleep as observed during the artificial conditions imposed by 104y-Gal4/UAS-TrpA1 activation.

Our multichannel data add to the growing realization that the entire insect brain engages in dynamical patterns of activity during both sleep and wake (*17*, *43*) and does not simply shut off when insects become immobile or quiescent. To understand these patterns of activity and how they might relate to conserved sleep functions (*50*) requires agnostic approaches derived from (for example) machine learning, as done in this study, rather than approximations inspired from human EEG.

## MATERIALS AND METHODS

### Animals

Flies (*D. melanogaster*) were reared on a standard fly medium under a 12-hour light/dark cycle (lights on at 8 a.m.). Flies were raised on a 25°C incubator (Tritech Research Inc.) with 50 to 60% humidity, and fewer than five flies were maintained per vial to ensure optimal nutrition and growth. Adult female flies (<3 days after eclosion) of wild-type Canton-S were used for the electrophysiological recordings. The choice of age of flies was based on pilot data that suggested a higher survival rate of younger flies over a 12-hour period on the air supported ball setup (after electrode insertion). Flies used for the behavioral dataset were between 3 and 7 days after eclosion. For thermogenetic experiments, refer to (*12*) for further details. No ethics committee approval was needed for all the studies.

### Fly tethering

First, flies were anesthetized on a thermoelectric cooled block maintained at a temperature of 1° to 2°C. Second, the thorax, dorsal surface, and wings of the fly were glued to a tungsten rod using dental cement (Coltene Whaledent SYNERGY D6 Flow A3.5/B3) and cured using high-intensity blue light (Radii Plus, Henry Schein Dental) for about 30 to 40 s. Further, dental cement was also applied to the necks to stabilize them and prevent lateral movement of the head during electrode insertion (see next section). Third, to prepare the fly for the multichannel overnight recording, we placed a sharpened fine wire made of platinum into the thorax (0.25 mm; A-M Systems). The platinum rod serves as a reference electrode and helps filter the noise originating from nonbrain sources. The insertion of a platinum electrode (while providing minimal discomfort to movement of animal) was done using a custom holder with a micromanipulator to enable targeted depth of insertion. For flies in the behavioral dataset, the procedure was the same, except that no reference wire was inserted.

### Multichannel preparation

First, the tethered fly from the previous step was placed on an air supported ball (polystyrene) that served as a platform for walking/resting. Humidified air was delivered to the fly using a tube below the ball (also from the side) to prevent desiccation. Second, to record from half of the regions in the fly brain (half-brain probe) we used a 16-electrode linear silicon probe (model no. A1x16-3 mm 25-177, NeuroNexus Technologies). Third, the probe was inserted into the eye of the fly laterally using a micromanipulator (Merzhauser, Wetzlar, Germany). The probe was inserted such that the electrode sites faced the posterior side of the brain. The final electrode position (depth of insertion) was determined using the polarity reversal procedure described below. For flies recorded in the behavioral dataset, the setup was similar, except that a custom chamber was lowered over the ball and fly to maintain a humidified environment during recordings.

### Polarity reversal

Variability in spatial location of recording sites across different flies is a primary impediment when comparing data across different flies. This occurs mainly because of the angle and depth of insertion of the probe, both of which cannot be precisely controlled. To overcome this issue and to obtain comparable recording sites across flies, we designed a paradigm using visual evoked potentials (fig. S2).

First, while the probe was being inserted from the periphery to the center of the brain, we used visual stimuli (square wave of 3 s in duration with 1-Hz frequency) from a blue light-emitting diode (LED). When the visual stimuli were displayed, we simultaneously recorded the LFPs from the 16 electrode sites. During the initial stage of insertion, most of the electrodes are outside of the brain, and only a few are inside the eye, optic lobe. The recordings in the electrodes inside the eye and the brain show a visual evoked potential corresponding to the leading edge and the trailing edge of the square wave. Second, we move the probe slowly toward the center of the brain so more of the electrode sites would now be inside the brain. Third, we notice that some electrodes have a negative deflection and some have a positive deflection with respect to the leading edge of the square wave. The electrodes in the eye, optic lobe regions, display a positive deflection, and electrodes further to the central parts of the brain display a negative deflection. However, this polarity change usually happens in the electrodes that are coincident on the regions right after the medulla. Fourth, for all flies, we made sure that the polarity change coincided with the electrodes 11 to 13 to establish consistency in terms of the spatial locations.

### Dye-based localization

To identify the possible locations in the brain targeted by the electrodes, we used a three-step procedure. In the first stage, we used immunohistochemistry to identify the locations of electrodes using a fluorescent dye and neuropils using antibodies against nc82 (presynaptic marker bruchpilot), respectively. In the second stage, we used a registration procedure to map the dye locations to an electron microscopy dataset (using nc82 images). In the third stage, we used principal components analysis to identify the precise neuropils targeted.

#### Immunohistochemistry

First, we labeled the probe with Texas red fluorescent dye conjugated to 10,000-Da molecular mass dextran dissolved in distilled water (Invitrogen) to identify the recording locations. Second, after removing the flies from the tether, the brains were dissected in 1× ice-cold phosphate-buffered saline (PBS) and fixed in 4% paraformaldehyde diluted in PBS-T (1× PBS and 0.2% Triton X-100) for 20 min in the dark to preserve the fluorescence of the dye. Third, after fixation, tissues were washed three times with PBS-T [with 0.01% sodium azide (Sigma-Aldrich)] and blocked for 1 hour in 10% goat serum (Sigma-Aldrich). Fourth, the brains were then incubated overnight in a primary antibody solution (mouse anti-nc82, Developmental Studies Hybridoma Bank; 1:20). Fifth, on the next day, brains were washed three times with PBS-T (10 min per wash) and incubated overnight in a secondary anti-body solution (1:250; goat anti-mouse Alexa Fluor 647). Last, the brain was washed in PBS-T and embedded in VECTASHIELD and imaged using a confocal microscope (Zeiss).

#### Image registration

First, for each fly, we used the nc82 image as source space to align to the JFRC2 template space [which is a spatially calibrated version of JFRC (*66*) from FlyLight]. The registration process involved two steps: (i) rigid affine registration that roughly aligned the source image to the template space with 12 degrees of freedom (translation, rotation, and scaling); and (ii) nonrigid registration that allowed different brain regions to move independently with a smoothness penalty. The entire process was carried out using the CMTK plugin (FiJi toolbox) as described here (*67*). Second, we then used the JFRC2 (light-level) registration as bridging registration to FAFB14 (electron microscopy dataset) using the natverse toolbox (*68*) and mapped both the nc82 images and the dye locations to the FAFB14 space.

#### Electrode localization

The electrode dye locations inside the brain are usually visible as fragments (points) instead of a single continuous (line) segment, mainly because the insertion of the probe causes the smearing of the dye on the neuropils in the brain. To identify the precise locations of the recording electrodes in the brain, we first used the points and performed principal component analysis to find the eigenvector or line (first principal component) that would have minimize the distance between the different points to the line itself. This line could be thought of as the main path of the probe as it entered into the brain. Next, we choose the innermost electrode as the projection of the innermost point (dye location) projected onto the eigenvector. The rest of the recording electrode sites were obtained by sampling the same eigenvector at intervals of 25 μm (which is the interelectrode distance on the probe) from the innermost point.

### LFP recording

The LFP data from the 16-electrode probe were acquired using Tucker-Davis Technologies (Tucker-Davis Technologies, USA) multichannel data acquisition system at 25 kHz coupled with a RZ5 Bioamp processor and RP2.1 enhanced real-time processor. Data were acquired and amplified using a preamplifier (RA16PA/RA4PA Medusa PreAmp). The preamplifier used can only record data of up to 20 hours on a single charge cycle; hence, we limited the recording of the LFP signals to 20-hour duration. Further, as file sizes tend to be larger over longer recording periods, we recorded data in chunks of 1 hour, which was automatically controlled via a MATLAB script.

### Video recording for flies on electrophysiology setup

The ball setup was illuminated with visible light, switched ON at 8 a.m. and switched OFF at 8 p.m. (mimicking the light/dark cycle conditions in the incubator). Further, we used infrared LEDs for monitoring the movement of the fly on the ball (which allowed us to quantify movements under both the light and the dark cycles. We recorded the fly in profile view with a digital camera from Scopetek (DCM 130E), and to achieve optical magnification, we used a zoom lens (from Navitar). As done previously (*12*), we removed the infrared filter in front of the camera sensor, to allow for filming under infrared light, thereby achieving constant illumination under both day and night. We made a custom script with Python (2.7.15) and OpenCV (3.4.2.17) that allowed for recording videos automatically and saving them in hourly intervals. The video was recorded with a resolution of 640 × 480 pixels at 30 frames/s using Xvid codec and further with additional metadata (time stamps in a csv file) that allowed a later matching up of the LFP data with the video data.

### Video recording for flies on behavioral dataset setup

The camera in this setup was a Point Grey/Teledyne FLIR Firefly perpendicular to the fly, in addition to an extra camera (Pro-MicroScan) placed on the trinocular output of a Nikon SZ7 stereomicroscope. This second camera was used to record a close-up view of the head of the fly for the purposes of tracking movements of the antennae. Illumination was as above with infrared LEDs, and recordings were obtained with the same Python scripts.

### Movement analysis

The fly movement was quantified with the video files using Python (3.6.1) and OpenCV (3.4.9) in the following manner. First, every video file (1 per hour of recording) was read frame by frame. Second, for each frame, we clipped the image such that the main focus was on the fly while ignoring items in the background. Third, we converted the color space for each frame from BGR to grayscale. Fourth, we computed the “deltaframe” as the absolute difference of the current frame with the previous frame. Fifth, we thresholded the deltaframe using a custom defined threshold per fly and converted them into binary. Sixth, we dilated the thresholded image and identified contours in the dilated image and looped over the different contours selecting those above a specific threshold (area). Last, we drew rectangles around the contours above the threshold on the original (color) image to manually verify the movement location. Only those frames that had contours above threshold were regarded as “moved” frames, and other frames would be classified as “still.” Thus, each frame would be either 0 (still) or 1 (moved).

In the next stage, we used the frame by frame movement data to identify segments of LFP data as sleep or awake in the following fashion. First, we synced the LFP data with the video data using the time stamps in both the LFP data and video metadata (csv files). Second, we clipped both the LFP and video data to the first 8 hours of recording. Though 23 flies survived for more than 12 hours, we only used the first 8 hours to ensure that the fly’s health was completely optimal (considering the circumstances) in both the behavior and brain recordings. Further, only 16 flies were used for the analysis, as 7 of them had issues with calibration (noisy or no calibration) or abnormal activity (either no sleep trials or very active). Third, we pruned movement data to ensure that brief noise in movements is avoided. Fourth, we identified the segments of data wherein the fly was immobile for more than 5 min as sleep and the segment immediately preceding 2 min before the sleep data as presleep and the rest of the data as awake.

### Crepuscular analysis

To identify whether the fly activity in our recordings followed a crepuscular pattern, first, we computed the movement pattern as proportion of frames moved per minute within these periods. Second, we divided the movement patterns across six different periods: (i) predawn: 5 to 7 a.m., (ii) dawn: 7 to 9 a.m., (iii) postdawn: 9 to 11 a.m., (iv) predusk: 5 to 7 p.m., (v) dusk: 7 to 9 p.m., and (vi) postdusk: 9 to 11 p.m. Third, we computed the *z* score of the movement pattern for normalization purposes, thus ending up with movement pattern per minute of the above mentioned time periods per fly.

### LFP analysis

#### Preprocessing

LFP data were analyzed with custom-made scripts in MATLAB (MathWorks) using EEGLAB toolbox (*69*). The preprocessing steps were as follows: First, the binary data were extracted for every hour from Tucker-Davis Technologies “tank” file format to MATLAB “mat” file format. Second, the data were resampled to 250 Hz and bandpass-filtered with zero phase shift between 0.5 and 40 Hz using hamming windowed-sinc FIR filter, and further line noise at 50 Hz was removed using a notch filter. Third, the hourly LFP data were saved to EEGLAB “.set” file format. Fourth, the hourly LFP data were interpolated in a linear way to avoid any discontinuities between the hourly segments of data. Fifth, the movement data (see the “Movement analysis” section) were added to the EEGLAB file along with the start and end time based on video data. Sixth, the multihour LFP data (along with the movement data) were collated for the first 8 hours of the recording. Seventh, we created separate epochs based on movement data into sleep, presleep, and awake [where preceding 2 min of immobility (−2 to 0 min) is presleep, immobility is sleep, and the rest of the data is awake, here, 0 min is the start of the immobility]. Eighth, the epochs were now re-referenced on the basis of the channel where the polarity reversal occurred. For this, we identified the channel wherein the polarity reversal occurred (see the “Polarity reversal” section) and subtracted all the channels from this channel, thus resulting in 15 channels after the re-referencing. This brain-based referencing technique (similar to the Cz-based reference in human EEG recordings) allows for filtering of nonbrain-based physiological noise components (such as heartbeat, etc.). Previous multichannel recordings used only the thorax-based referencing (followed by bipolar referencing) along with independent component analysis to remove physiological noises. However, the identification of noise components such as heartbeat, etc. from independent component analysis is subjective and further depends on the expertise of the human curator. Our technique overcomes these issues while simultaneously providing a method to remove physiological noises not originating from the brain.

#### Power spectrum analysis (sleep versus wake)

The power spectra of the LFP data were computed for each fly in the following fashion. First, each condition (“wake” and sleep) of varying duration was reepoched into trials of 60-s duration. Second, each trial was bandpass-filtered with zero phase shift between 5 and 40 Hz using hamming windowed-sinc FIR filter. Third, for each trial, power spectra (in decibels) were computed using the “spectopo” function in the EEGLAB toolbox in MATLAB. Fourth, the mean power spectra for all the trials per condition per fly were computed. The goal of the power spectra analysis was to identify the cluster of frequency bands and channels that differ across the sleep and wake periods at the group level. To perform these group level comparisons (sleep versus wake periods), we only used flies that had at least 10 trials under each condition. To identify the differences across wake and sleep periods, we used cluster-based permutation tests. Cluster-based permutation tests (*70*) are a nonparametric way of testing difference across conditions in an *N*-dimensional space (here, frequencies × channels) while still allowing for the multiple comparison problems to be solved without reducing the statistical power of the test. The outcome of such a test would be significant cluster(s), which, in our case, would be an ROI across frequencies × channels. Thus, we performed a cluster permutation test (flies × frequencies × channels) using MNE (0.22.0) in Python (permutation_cluster_1samp_test) (*71*) with all possible permutations to identify clusters (ROIs in frequencies × channel space) that differ across awake and sleep periods. We also computed the effect sizes for every channel × frequency combination using Cohen’s *d* measure (difference of means/SD).

### Thermogenetic sleep induction

The thermogenetic sleep induction data were collected using 104y-Gal4/UAS-TrpA1 lines as part of the study (*12*). This multichannel recording consisted of a 16-electrode full-brain probe (model no. A1x16-3 mm50-177, NeuroNexus Technologies) covering the whole of the brain (fig. S7B) (in contrast to the half-brain probe mentioned before) with an interelectrode distance of 50 μm. The rest of the recording parameters were the same as mentioned in the previous section. Sleep induction was achieved by transient activation of this circuit, as described in (*12*).

#### Preprocessing

LFP data were analyzed with custom-made scripts in MATLAB (MathWorks) using EEGLAB as mentioned before. The preprocessing steps were as follows: First, the LFP data per condition (“baseline,” “sleep induction,” and “recovery”) were converted to EEGLAB .set file format with a sampling rate of 1 kHz. Second, the LFP data were re-referenced using a differential approach, wherein nearby channels are subtracted with each other resulting in 15 channels.

#### Power spectrum analysis (baseline versus sleep induction)

The power spectra of the LFP data were computed for each fly in the following fashion. First, each condition (baseline and sleep induction) was reepoched into trials of 1-s duration. Second, each trial was bandpass-filtered with zero phase shift between 5 and 40 Hz using hamming windowed-sinc FIR filter. Third, for each trial, power spectra (in decibels) were computed using the spectopo function in the EEGLAB toolbox in MATLAB. Fourth, the mean power spectra for all the trials per condition per fly were computed. The group level comparison was performed using cluster permutation test methods (as described in previous sections) to identify differences in frequency × channels across baseline and sleep induction conditions.

### Sleep staging by classifiers

The main goal of this analysis was to use classifiers to identify the existence of sleep stages using LFP data.

#### Labeling of sleep states

Here, we relabeled the segments of data (already identified as sleep and awake based on movement data) in the following fashion. First, we labeled the segments of data in the first 2 min (0 to 2 min) after the start of immobility as earlysleep and the segments of the data in the preceding 2 min (−2 to 0 min) as presleep. Second, we labeled the segments of data in the last 2 min of sleep as latesleep and the segments of data in between the earlysleep and latesleep as midsleep. The rest of the data are considered as awake.

#### Preprocessing and power spectrum computation

The preprocessing steps were the same as mentioned in the previous section (LFP preprocessing). For the computation of the power spectrum, we followed similar procedures as mentioned before; however, we saved the individual power spectrum per trial (channels × frequency) per fly in a csv file along with the corresponding label of the sleep state.

#### Classifier probability analysis

We implemented an SVM-based classifier using scikit-learn (0.24.2) to classify the LFP data using the following steps. First, we collated the features based on power spectrum (channels × frequency) from all the flies across different sleep states. Second, we filtered the features to only awake (5106 epochs) and midsleep (1165 epochs) states. Here, we also did not feed (for training) the preceding 2 min of presleep, succeeding 2 min of earlysleep, and the last 2 min of sleep latesleep into the classifier (we used those minutes for sanity check purposes; refer to Fig. 5A). Third, we encoded the target labels (awake and midsleep) into binary states using “LabelEncoder” from scikit-learn. Fourth, we balanced the composition of labels (or classes) to prevent bias due to unequal distribution of classes in the training dataset. Fifth, we divided the dataset into train and test sets (80% train and 20% test) using “train_test_split” from scikit-learn in a stratified fashion. Sixth, we subjected both the train and test data to a standard scaler using “StandardScaler” from scikit-learn, which removes the mean of the data and scales it by the variance. Seventh, we implemented an SVM-based classifier using a “linear” kernel along with probability estimates per class and fit the classifier to the train dataset. Eighth, we used the trained classifier on the test dataset and computed different metrics of classifier performance such as accuracy, roc_auc, recall, precision, and F1 score using “metrics” from scikit-learn (fig. S8B). Ninth, we used the trained classifier on all class labels (awake, presleep, earlysleep, midsleep, latesleep, preceding 2 min of presleep, and succeeding 2 min of latesleep) from the original dataset and computed the probability estimates per class. Note that none of the presleep, earlysleep, latesleep, preceding 2 min of presleep, and succeeding 2 min of latesleep data have not been seen by the classifier beforehand. The above process from step 5 onward is repeated a further four times with different test and train splits to create five different iterations of classifiers and performance metrics.

#### Multiclass random forest classifier analysis and feature importance

To identify differences across multiple classes (awake, presleep, earlysleep, midsleep, and latesleep), we implemented a random forest classifier using scikit-learn (0.24.2) to classify the LFP data using the following steps. First, we collated the features based on power spectrum (channels × frequency) from all the flies across different sleep states. Second, as the different labels (or classes) were unbalanced for: awake (5585 epochs), presleep (258 epochs), earlysleep (262 epochs), midsleep (1165 epochs), and latesleep (262 epochs), we used SMOTE (synthetic minority oversampling technique) from imblearn (0.8.1) to balance the distribution of classes in the dataset. Third, we divided the dataset into train and test sets (80% train and 20% test) using train_test_split from scikit-learn in a stratified fashion. Fourth, we subjected both the train and test data to a standard scaler using StandardScaler from scikit-learn, as mentioned in the previous section. Fifth, we encoded the target labels into binary states using “LabelBinarizer” from scikit-learn. Sixth, we implemented a random forest classifier for this multiclass classification problem. As the random forest classifier has multiple hyperparameters that need to be tuned, we first used a random grid (using “RandomizedSearchCV” from scikit-learn) to search for the hyperparameters and then further used these parameters in a grid search model (using “GridSearchCV” from scikit-learn) to identify the best hyperparameters. Seventh, we used the trained classifier on the test dataset and computed different metrics of classifier performance such as recall, precision, F1 score, etc. using metrics from scikit-learn separately for all the five classes. Furthermore, we also computed a normalized confusion matrix using “confusion_matrix” from scikit-learn. The above process from step 5 onward is repeated a further four times with different test and train splits to create five different iterations of classifiers and performance metrics. Last, to identify and rank the importance of different features, we used the permutation importance metric (using “permutation_importance” from scikit-learn). The permutation feature importance works by randomly shuffling a single feature value and further identifying the decrease in the model score (*72*). The process breaks the relationship between the shuffled feature and the target; thus, if the feature is very important, it would be indicated by a high drop in model score; on the other hand, if it is relatively unimportant, then the model score would not be affected so much. We used the permutation importance with a repeat of 5, and for each train/test split, we computed a permutation importance score. Last, the mean permutation importance score was computed using all the splits. The procedure for differentiating across daysleep and nightsleep periods was the same except the target classification was across daysleep (917 epochs) and nightsleep (770 epochs) classes.

#### Classifier metrics

The performance of the abovementioned classifiers (both SVM-based and random forest–based) was evaluated using metrics such as accuracy, recall, precision, roc_auc, and F1 score. The definitions of these metrics are as follows:

1) Recall: This refers to the ability of a classifier to correctly detect the true class of the epoch among the classifications made. It is obtained by the (TP/TP + FN). It is also known as sensitivity. TP indicates true positives, and FN indicates false negatives.

2) Precision: This refers to the exactness of the classifier. It is obtained by the (TP/TP + FP).

3) F1 score: This refers to the harmonic mean between precision and recall.

4) roc_auc: This refers to the area under the receiver operating curve. In general, it refers to how efficient the classifier is in identifying different epochs. Scores closer to 1 indicate a highly efficient classifier, whereas those closer to 0 indicate otherwise.

5) Accuracy: This is defined as the number of correctly classified epochs divided by the overall number of epochs classified.

6) Confusion matrix: This enables visualization of the classifier performance, by tabulating the predicted classes against actual classes. For multiclass problems (random forest classifiers here), the values in the diagonal indicate where the predicted and actual classes converge, whereas those on the off-diagonal indicate misclassifications.

### Proboscis tracking for flies on electrophysiology setup

#### Pose detection

We used DeepLabCut (*30*) to track the different body parts of the fly using an artificial neural network trained in the following fashion. First, we extracted frames from sample videos, wherein the fly performs the following: normal walking movement on the ball (“all_body”) and PE periods (“proboscis”), both while asleep and awake. For each fly, we extracted videos of the abovementioned categories for the purpose of creating annotation labels. Second, we extracted frames from these videos and further labeled the different body parts: eye, proboscis, leg1_tip, leg1_joint, leg3_tip, leg3_joint, and abdomen (Fig. 7A). Third, we trained the neural network per fly using this dataset with “resnet_50” weights until the loss parameter during training stabilizes. The performance of the network per fly (train and test error in pixels) was in general similar in both the train and test datasets. Fourth, we evaluated the annotation performance manually by labeling a test video and verifying the same. Last, this trained network (per fly) was used for annotating the video for the first 9 hours of the recording.

#### Pose analysis

In the next step, we use the pose detection output to design a classifier capable of identifying PE periods. First, we manually detected several sample time points (to be used as ground truth for training/testing the classifier) in the video of each fly, identified proboscis time periods, and saved them in a “csv” file. Second, we used the pose tracking data (*x*,*y* likelihood) for the body parts of the proboscis, leg1_tip, leg1_joint, eye, and abdomen and further computed low pass–filtered data (0.1-Hz butterworth filter) of each body part. Further, we also computed the moving average (window length of five samples) of the filtered data. Third, we computed “dist_eyeprob” as the Euclidean distance between the proboscis and eye body part and lastly multiplied the same with the likelihood of the proboscis body part. Fourth, we used the abovementioned body parts (and its derivatives) as features and used the StandardScaler from scikit-learn for normalizing the data. Fifth, we divided the dataset into train and test sets (70% train and 30% test) using train_test_split from scikit-learn. Sixth, we implemented an SVM-based classifier using an “rbf” kernel and fit the classifier to the train dataset. Seventh, we used the trained classifier on the test dataset and computed different metrics of classifier performance such as accuracy, recall, precision, etc. using metrics from scikit-learn. The data segments (frames) identified here will be used to construct the candidate proboscis periods, which then will be further refined in the next steps.

#### Proboscis detection

First, we use the frames identified by the classifier from the previous section and construct continuous segments to identify time periods of probable proboscis periods. Further, we add additional time periods using the likelihood of the proboscis part with a threshold-based method. Second, we identify the peak frame (where the maximum displacement of the proboscis occurs) in each PE event (each proboscis bout consists of multiple PE events) and save the identified proboscis events (frame number, time, and behavior state) to a csv file. Third, each event in the csv file is manually verified, and only true events are further taken forward. This process is repeated for all the flies, and the proboscis detection accuracy per fly is plotted in Fig. 7C.

### Microbehavior tracking for flies on behavioral dataset setup

Here, the same method for tracking microbehaviors via DeepLabCut was used, focusing on the proboscis and abdomen for the lateral camera view (see above) and the base and tip of the left and right antennae for the dorsal view of the fly head. The data from these two streams were imported into a custom MATLAB (2020a) script, which performed synchronization based on the integrated time stamps. After preprocessing, antennal tracking with DeepLabCut was converted into an angle for both respective antennae by calculation of the respective positions of the bases and tips, with the angle of the fly’s head with respect to the camera automatically derived from these data and used to correct the angle of the antennae. For the proboscis, a median position was calculated for each recording—assumed to be the resting position—and the distance and angle between the proboscis at any given time point, and this median position was calculated. Extensions of the proboscis were derived from these distance data with the “findpeaks” function in MATLAB, with a number of exclusion criteria applied to remove tracking artifacts. For example, detected peaks that exceeded a biologically plausible distance threshold, lasted only for a single frame, or had an implausible instantaneous rise time were excluded. Since this method could potentially be biased toward identifying proboscis activity that follows a prototypical shape, we also used an alternative proboscis event detection based purely on the current distance of the proboscis from resting. In this, we used a manually set threshold for each fly to detect portions in the recording when the proboscis was extended versus not, and for these “events,” we calculated the duration and median angle of the proboscis during the span of the event. Periods of antennal periodicity in recordings were calculated on the basis of a fast Fourier transform and applied to time segments of recordings. Since proboscis activity was not sinusoidal in nature (and thus would behave poorly if subjected to a fast Fourier transform), periodicity for this organ was calculated manually as a factor of timing between individual PEs in that PEs were periodic if they occurred less than 6 s after a preceding PE. This value was selected from observation of typical inter-PE intervals.

### LFP analysis—Proboscis

The main goal of this analysis was to identify the spectral signatures associated with the PE periods across awake and sleep states in the LFP data.

#### Identification of proboscis periods

First, we used the csv file containing frame by frame detection of manually verified proboscis events (from the section above). Second, we identify periods of PEs which are close together (within 10 s of each other) and label them as continuous periods. Third, we add activity labels such as awake (awake periods without any proboscis activity), “awakeprob” (awake periods with proboscis activity), sleep (sleep periods without any proboscis activity), “sleepprob” (sleep periods with proboscis activity), presleep (presleep periods without any proboscis activity), and “presleepprob” (presleep periods with proboscis activity) based on annotated behaviors. Fourth, we extract the LFP data corresponding to the different time periods across each fly.

#### Power spectrum analysis

The preprocessing steps for the extracted LFP data were the same as mentioned in the previous section (LFP preprocessing). For the computation of the power spectrum, we followed similar procedures as mentioned before; however, we computed the individual power spectrum per trial (channels × frequency) per fly by reepoching them into trials of 1 s in duration (instead of the 60-s periods for sleep analysis, as the proboscis periods are usually shorter). Then, the mean power spectrum for all the trials per condition per fly was computed. Next, we performed cluster permutation tests (flies × frequencies × channels) for identifying the differences across frequencies and channels across different conditions. For this analysis we only used flies that had at least 50 trials under each condition.

### Multilevel models

#### Models for antennal and proboscis periodicity

We defined two different multilevel models (tables S1, S3, and S5, left and right antenna and proboscis) to understand how the likelihood of periodicity varies by sleep epoch. In the null model, the periodicity depends only on the mean per fly (fixed effect) and the fly ID (random effect). In the second model (epoch model), the periodicity depends only on the epoch (fixed effect) and the fly ID (random effect). These models were fit using the “lmer” function (“lmerTest” package) in R (*73*), and the winning model is identified as the one with the highest log-likelihood by comparing it with the null model and performing a likelihood ratio chi-square test (χ^2^). Last the winning model was analyzed using the “anova” function (tables S2, S4, and S6, left and right antenna and proboscis) in R (*74*).

#### Models for movement pattern across crepuscular periods

We defined two different multilevel models separately for dawn and dusk periods (tables S7 and S9, movement pattern in dawn and dusk periods) to understand how the movement pattern of the flies varies by different twilight hours. In the null model, the movement depends only on the mean per fly (fixed effect) and the fly ID (random effect). In the second model (crepuscular-type model), the movement depends only on the crepuscular-type (fixed effect) and the fly ID (random effect). These models were fit using the lmer function (lmerTest package) in R (*73*), and the winning model is identified as the one with the highest log-likelihood by comparing it with the null model and performing a likelihood ratio chi-square test (χ^2^). Last, the winning model was analyzed using the anova function (tables S8 and S10, movement pattern in dawn and dusk periods) in R (*74*).

#### Models for movement pattern across recorded hours

We defined two different multilevel models (table S11, movement pattern across recorded hours) to understand how the movement of the flies (thereby health) varies by different recording hours. In the null model, the movement depends only on the mean per fly (fixed effect) and the fly ID (random effect). In the second model (recorded hour model), the movement depends only on the recorded hour (fixed effect) and the fly ID (random effect). These models were fit using the lmer function (lmerTest package) in R (*73*), and the winning model is identified as the one with the highest log-likelihood by comparing it with the null model and performing a likelihood ratio chi-square test (χ^2^). Last, the winning model was analyzed using the anova function (table S12, movement pattern across recorded hours) in R (*74*).

#### Models for LFP power spectrum across recorded hours

We defined two different multilevel models separately for awake and sleep periods (tables S13 and S15, LFP power spectrum in awake and sleep periods across recorded hours) to understand how the different recording hours (thereby consistency of recordings) affected the LFP power spectrum. In the null model, the LFP power spectrum depends only on the mean per fly (fixed effect) and the fly ID (random effect). In the second model (recorded hour model), the LFP power spectrum depends only on the recorded hour (fixed effect) and the fly ID (random effect). These models were fit using the lmer function (lmerTest package) in R (*73*), and the winning model is identified as the one with the highest log-likelihood by comparing it with the null model and performing a likelihood ratio chi-square test (χ^2^). Last, the winning model was analyzed using the anova function (table S14, LFP power spectrum in awake periods across recorded hours; for the sleep periods, the winning model was the null model, so was not analyzed further) in R (*74*).

#### Models for spectral analysis

We defined four different multilevel models (table S16) to understand the modulation of the power spectrum by sleep epoch and channel type. In the null model, the power spectrum depends only on the mean per fly (fixed effect) and the fly ID (random effect). In the second model (epoch model), the power spectrum depends only on the LFP epoch type (fixed effect) and the fly ID (random effect). In the third model (channel model), the power spectrum depends only on the channel type (fixed effect) and the fly ID (random effect). In the fourth model (epoch channel model), the power spectrum depends on a combination of the LFP epoch type and the channel type, both used as fixed effects and the fly ID (random effect). These four models were fit using the lmer function (lmerTest package) in R (*73*), and the winning model is identified as the one with the highest log-likelihood by comparing it with the null model and performing a likelihood ratio chi-square test (χ^2^). Last, the top two winning models were compared against each other using anova function in R (*74*), to validate whether the winning model (if it is more complex) is actually better than the losing model (if it is simpler). The epoch channel model emerged as the winning model, indicating an important contribution from different channels. The epoch channel was further analyzed with the anova function (table S17) in R (*74*).

#### Models for PE event counts

We defined two different multilevel models (table S18) to understand the modulation of PE event count by sleep epochs. In the null model, the PE event count depends only on the mean per fly (fixed effect) and the fly ID (random effect). In the second model (time_label model), the PE event count depends only on the specific temporal sleep stage (fixed effect) and the fly ID (random effect). These two models were fit using the lmer function (lmerTest package) in R (*73*), and the winning model is identified as the one with the highest log-likelihood by comparing it with the null model and performing a likelihood ratio chi-square test (χ^2^). Thus, the time_label model emerged as the winning model. The time_label model was further analyzed with the anova function (table S19) in R (*74*).
