# Supplementary material for: Multivariate classification of multichannel long-term electrophysiology data identifies different sleep stages in fruit flies
Source: Sci Adv. 2024 Feb 21;10(8):eadj4399. doi: 10.1126/sciadv.adj4399 (PMC10881036; doi:10.1126/sciadv.adj4399)
Supplement: Supplementary file 1 — Tables S1 to S19 Figs. S1 to S9 [file sciadv.adj4399_sm.pdf]

Science Advances
AAAS

Supplementary Materials for

Multivariate classification of multichannel long-term electrophysiology data identifies different sleep stages in fruit flies

Sridhar R. Jagannathan *et al.*

Corresponding author: Sridhar R. Jagannathan, sridhar-rajan.jagannathan@charite.de; Bruno van Swinderen, b.vanswinderen@uq.edu.au

Sci. Adv. **10**, eadj4399 (2024)
DOI: 10.1126/sciadv.adj4399

This PDF file includes:

Tables S1 to S19
Figs. S1 to S9

Supplementary material

Suppl Table 1: Model comparison - Left antenna

**Model**	**Parameters**	**Log-likelihood**	**Pr(>χ2)**
Null	Fixed: mean, Random: fly ID	-106.53	-
epoch	Fixed: time_label, Random: fly ID	**-73.58**	<0.001

Suppl Table 2: Type III analysis of variance with Satterthwaite's method of the winning model (Epoch) - Left antenna

**Model elements**	**Sum Sq**	**Mean Sq**	**NumDF**	**DenDF**	**F value**	**Pr(>F)**
Epoch	4.5527	1.1382	4	1090	16.985	<0.001

Suppl Table 3: Model comparison - Right antenna

**Model**	**Parameters**	**Log-likelihood**	**Pr(>χ2)**
Null	Fixed: mean, Random: fly ID	-68.415	-
epoch	Fixed: time_label, Random: fly ID	**-44.468**	<0.001

Suppl Table 4: Type III analysis of variance with Satterthwaite's method of the winning model (Epoch) - Right antenna

**Model elements**	**Sum Sq**	**Mean Sq**	**NumDF**	**DenDF**	**F value**	**Pr(>F)**
Epoch	3.1004	0.7751	4	1125	12.232	<0.001

Suppl Table 5: Model comparison - PEs

**Model**	**Parameters**	**Log-likelihood**	**Pr(>χ2)**
Null	Fixed: mean, Random: fly ID	-301.09	-

epoch	Fixed: time_label, Random: fly ID	**-207.25**	<0.001

Suppl Table 6: Type III analysis of variance with Satterthwaite's method of the winning model (Epoch) - PEs

**Model elements**	**Sum Sq**	**Mean Sq**	**NumDF**	**DenDF**	**F value**	**Pr(>F)**
Epoch	20.877	5.2192	4	795	52.923	<0.001

Suppl Table 7: Model comparison – movement pattern in dawn periods

**Model**	**Parameters**	**Log-likelihood**	**Pr(>χ2)**
Null	Fixed: mean, Random: fly ID	-1172.9	-
Crepuscular-type	Fixed: crepuscular-type, Random: fly ID	**-1167.5**	<0.01

Suppl Table 8: Type III analysis of variance with Satterthwaite's method of the winning model (crepuscular-type) – movement pattern in dawn periods

**Model elements**	**Sum Sq**	**Mean Sq**	**NumDF**	**DenDF**	**F value**	**Pr(>F)**
Crepuscular-type	16.066	8.0329	2	29.878	7.8492	<0.01

Suppl Table 9: Model comparison – movement pattern in dusk periods

**Model**	**Parameters**	**Log-likelihood**	**Pr(>χ2)**
Null	Fixed: mean, Random: fly ID	-4422.5	-
Crepuscular-type	Fixed: crepuscular-type, Random: fly ID	**-4347.3**	<0.001

Suppl Table 10: Type III analysis of variance with Satterthwaite's method of the winning model (crepuscular-type) – movement pattern in dusk periods

**Model elements**	**Sum Sq**	**Mean Sq**	**NumDF**	**DenDF**	**F value**	**Pr(>F)**

Crepuscular-type	140.25	70.123	2	3021	78.131	<0.001

Suppl Table 11: Model comparison – movement pattern across recorded hours

**Model**	**Parameters**	**Log-likelihood**	**Pr(>χ2)**
Null	Fixed: mean, Random: fly ID	-76.525	-
Recorded-hours	Fixed: recorded-hours, Random: fly ID	**-64.886**	<0.01

Suppl Table 12: Type III analysis of variance with Satterthwaite's method of the winning model (recorded-hours) – movement pattern across recorded hours

**Model elements**	**Sum Sq**	**Mean Sq**	**NumDF**	**DenDF**	**F value**	**Pr(>F)**
Recorded-hours	4.1198	0.58854	7	128	3.6471	<0.01

Suppl Table 13: Model comparison – LFP power spectrum in awake periods across recorded hours

**Model**	**Parameters**	**Log-likelihood**	**Pr(>χ2)**
Null	Fixed: mean, Random: fly ID	-5936.5	-
Recorded-hours	Fixed: recorded-hours, Random: fly ID	**-5934.3**	<0.05

Suppl Table 14: Type III analysis of variance with Satterthwaite's method of the winning model (recorded-hours) – LFP power spectrum in awake periods across recorded hours

**Model elements**	**Sum Sq**	**Mean Sq**	**NumDF**	**DenDF**	**F value**	**Pr(>F)**
Recorded-hours	120.74	120.74	1	1904	4.3687	<0.05

Suppl Table 15: Model comparison – LFP power spectrum in sleep periods across recorded hours

**Model**	**Parameters**	**Log-likelihood**	**Pr(>χ2)**

Null	Fixed: mean, Random: fly ID	-4005.6	-
Recorded-hours	Fixed: recorded-hours, Random: fly ID	-4005.3	>0.05

Suppl Table 16: Model comparison - LFP power spectrum

**Model**	**Parameters**	**Log-likelihood**	**Pr(>χ2)**
Null	Fixed: mean, Random: fly ID	-68117	-
Epoch	Fixed: epoch, Random: fly ID	-67941	<0.001
Channel	Fixed: channel, Random: fly ID	-52391	<0.001
Epoch-Channel	Fixed: epoch*channel, Random: fly ID	**-51593**	<0.001

Suppl Table 17: Type III analysis of variance with Satterthwaite's method of the winning model (Epoch-Channel) - LFP power spectrum

**Model elements**	**Sum Sq**	**Mean Sq**	**NumDF**	**DenDF**	**F value**	**Pr(>F)**
Epoch	8476	2119	4	22582	378.025	<0.001
Channel	112004	56002	2	22580	9990.441	<0.001
Epoch:Channel	796	100	8	22580	17.756	<0.001

Suppl Table 18: Model comparison - PEs LFP dataset

**Model**	**Parameters**	**Log-likelihood**	**Pr(>χ2)**
Null	Fixed: mean, Random: fly ID	-4.5588	-
time_label	Fixed: time_label, Random: fly ID	**15.0632**	<0.001

Suppl Table 19: Type III analysis of variance with Satterthwaite's method of the winning model (time_label) - PEs LFP dataset

Model elements	Sum Sq	Mean Sq	NumDF	DenDF	F value	Pr(>F)
time_label	1.2145	0.20241	6	41	9.6039	<0.001

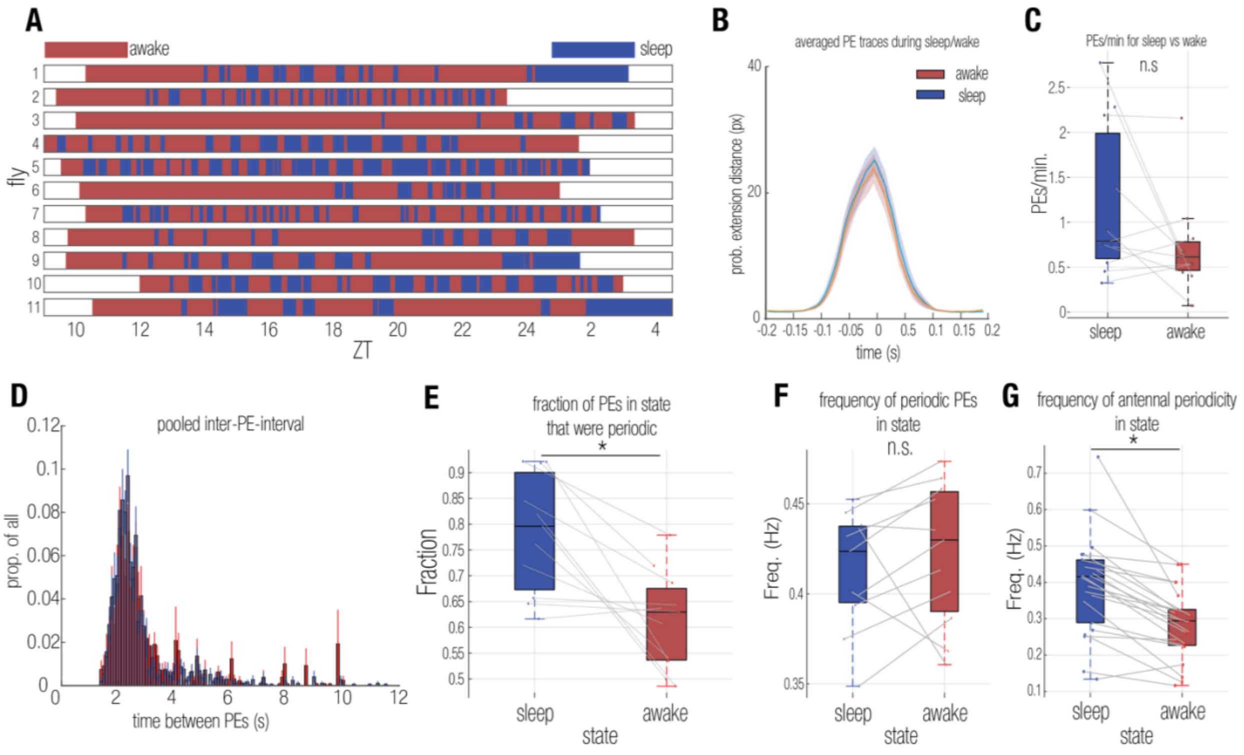

Supplementary Figure 1: Additional metrics of proboscis activity during sleep and wake. A) Representation of the distribution of sleep (Blue) and wake (Red) across N=11 recorded individuals over the course of time. B) Averaged timecourse of proboscis extension distance from resting during a single event for sleep (Blue) and wake (Red). C) Comparison of proboscis extension rates during sleep and wake (n.s. ; Student's T-test). D) Histogram of the distribution of times between PEs during sleep (Blue) and wake (Red). E) Comparison of the fraction of PEs that were periodic versus isolated for sleep and wake ($p < 0.05$; Student's T-test). F) Comparison of the average frequency of PE periodicity across sleep and wake (n.s.; Student's T-test). G) As with F, for antennal periodicity ($p < 0.05$; Student's T-test).

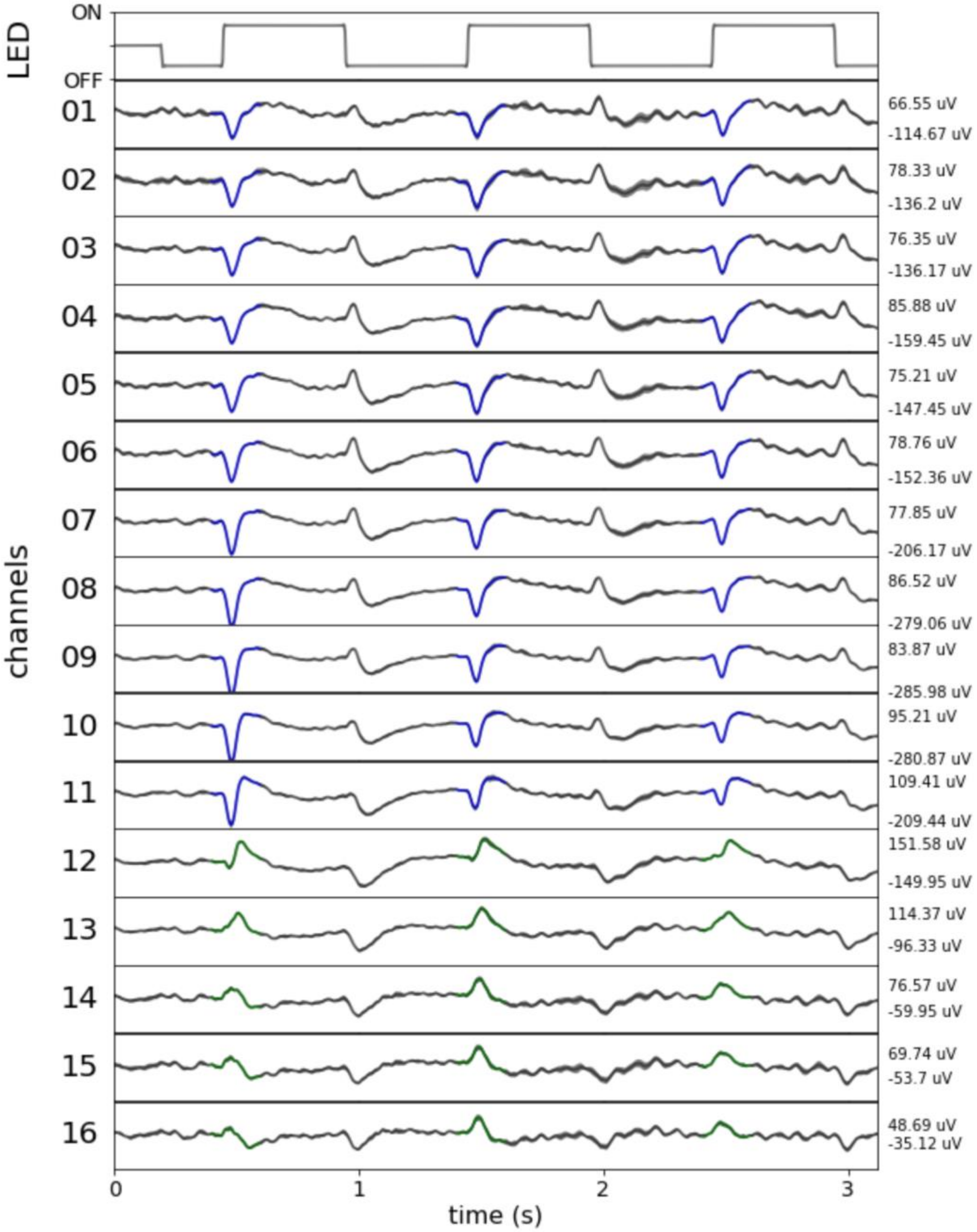

Supplementary Figure 2: Polarity reversal across an example fly. Electrode insertion depth was controlled by using a polarity reversal method. In this example fly, the change in LED stimulation (OFF to ON) stage, coincides with a LFP deflection. The LFP deflection changes from positive (12th channel) to negative (11th channel). The LFP amplitude depicted here is based on an average of 5 trials, with the shaded region representing the standard error.

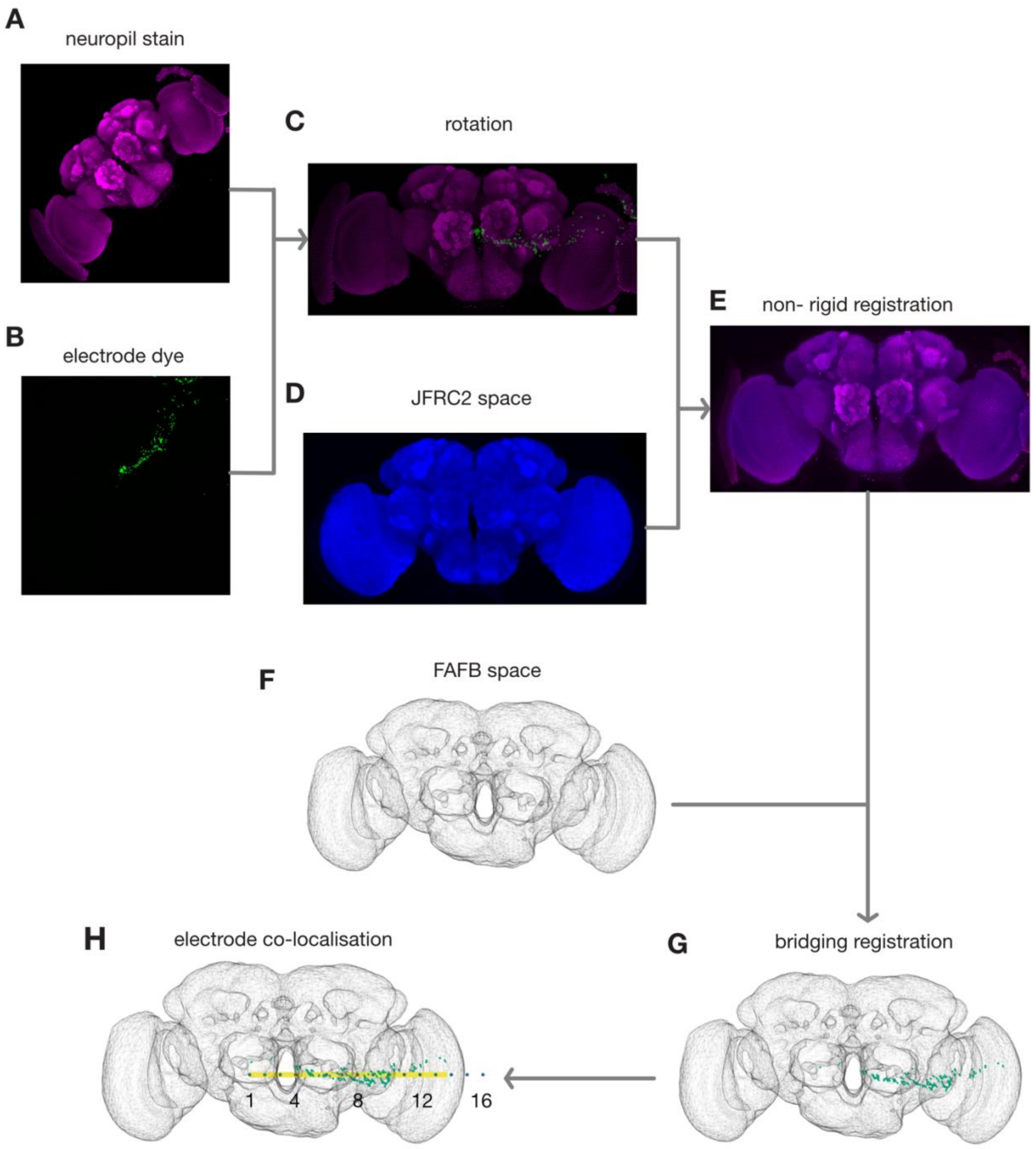

Supplementary Figure 3: Electrode location identification in an example fly. Electrode locations were determined using a dye-based localization method. Neuropil stain (A) and electrode dye locations (B) were registered to JFRC2 space (D) via non-rigid registration. Neuropil stain (A) and electrode dye locations (B) are rotated for co-visualization purposes (C). Depiction of neuropil stain (A) post-transformation to JFRC2 space (E). Further Bridging registration was used to register to FAFB space (F), the registration templates were applied on electrode dye locations (B) to produce co-localization (G) and further depicted with electrode locations (H) based on principal component analysis.

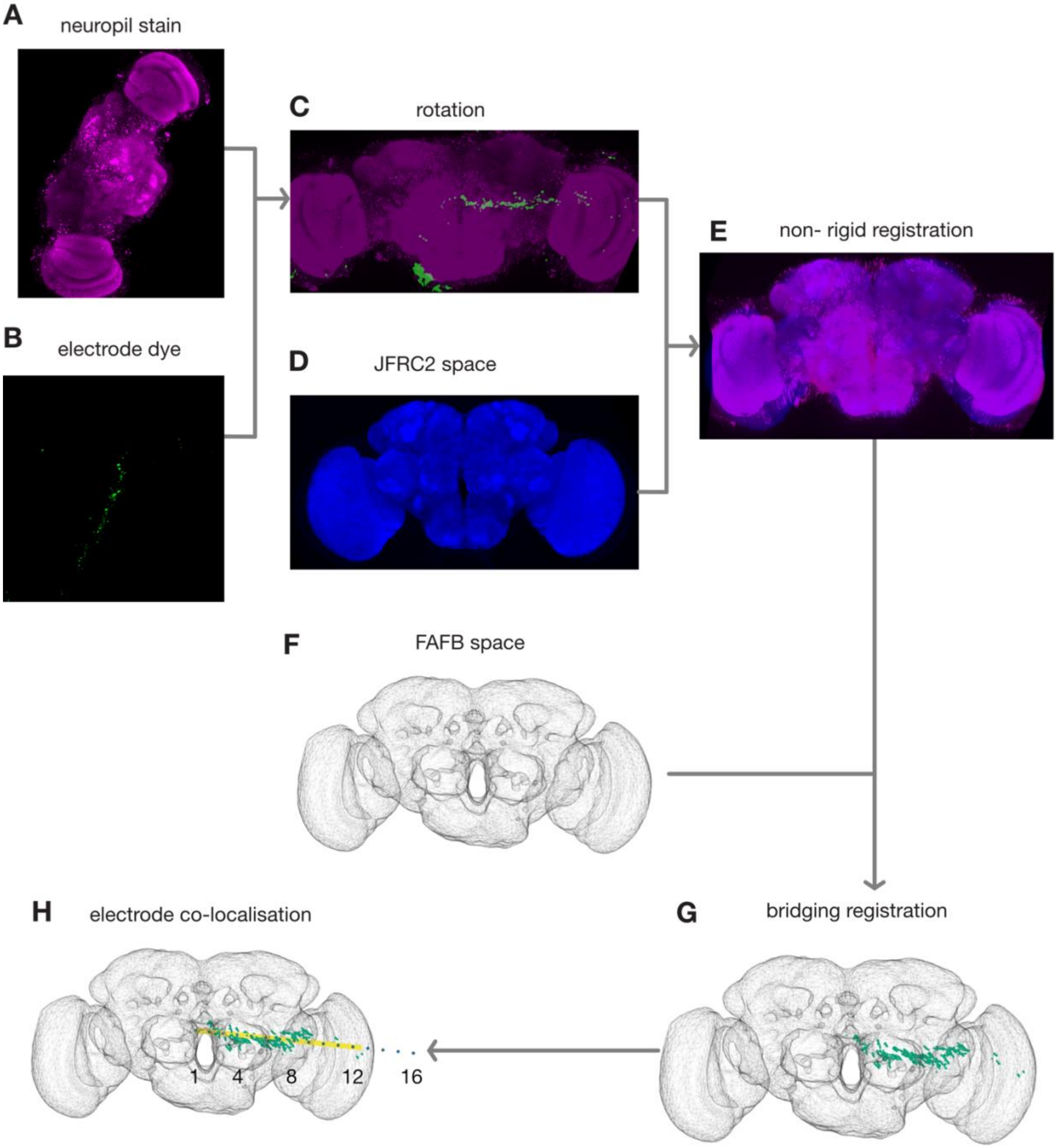

Supplementary Figure 4: Electrode location identification in an example fly. Electrode locations were determined using a dye-based localization method. Neuropil stain (A) and electrode dye locations (B) were registered to JFRC2 space (D) via non-rigid registration. Neuropil stain (A) and electrode dye locations (B) are rotated for co-visualization purposes (C). Depiction of neuropil stain (A) post-transformation to JFRC2 space (E). Further Bridging registration was used to register to FAFB space (F), the registration templates were applied on electrode dye locations (B) to produce co-localization (G) and further depicted with electrode locations (H) based on principal component analysis.

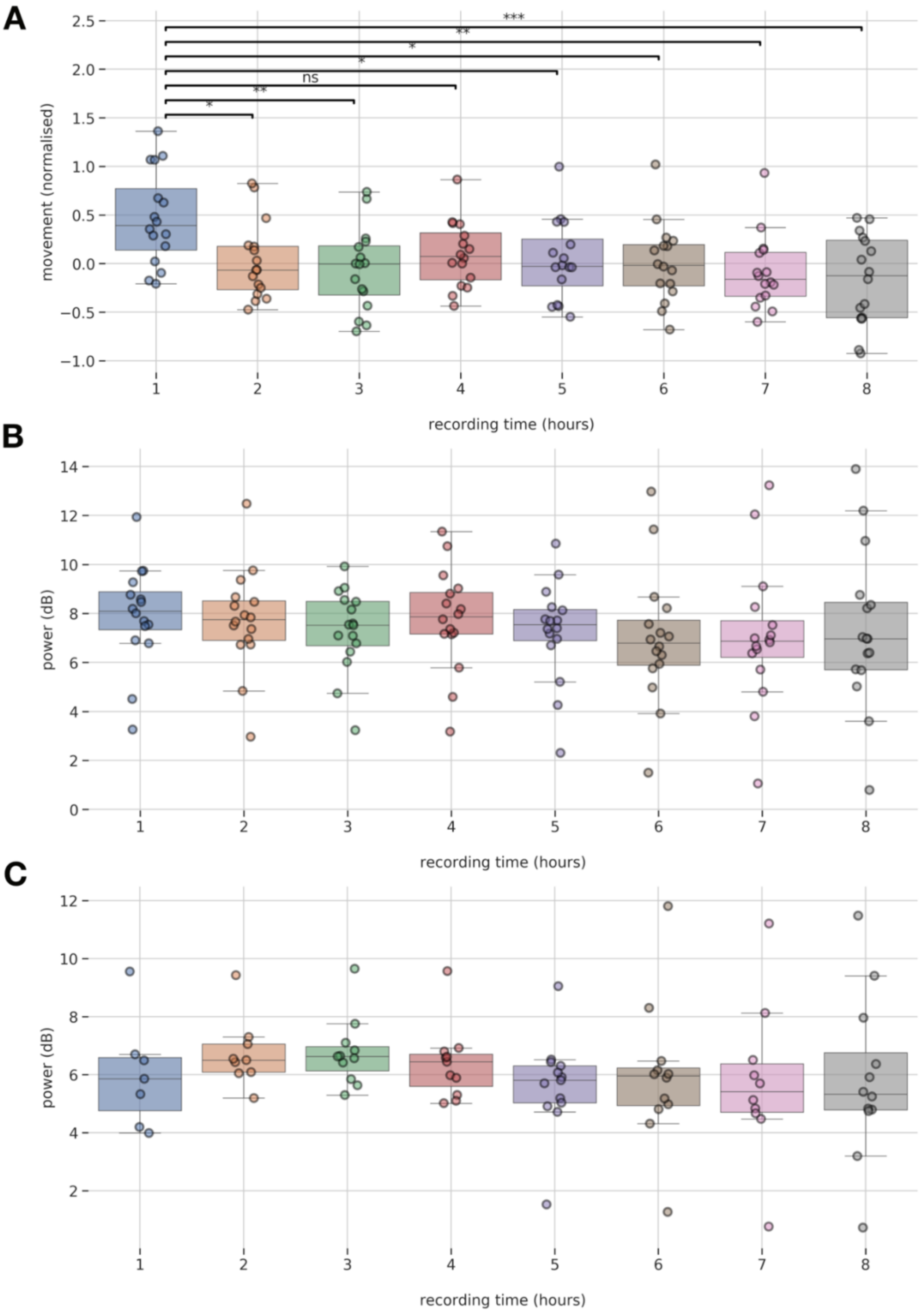

Supplementary Figure 5: Cross-validation of fly health (using movement pattern), recording consistency (using awake, sleep LFP power) across recorded hours. A) Movement (activity pattern) of different flies across recording hours. Only the first hour of recording differs from most of the rest, the subsequent hours are similar to each other. *p<0.05, **p<0.01,***p<0.001, ns indicates not significant. B) Average power spectrum during awake periods of different flies across recording hours. The different recording hours doesn't differ in the average power of awake

periods, indicating qualitatively the lfp recordings do not change over the recording hours (1-8). C) Average power spectrum during sleep periods of different flies across recording hours. The different recording hours doesn't differ in the average power of sleep periods, indicating qualitatively the lfp recordings do not change over the recording hours (1-8).

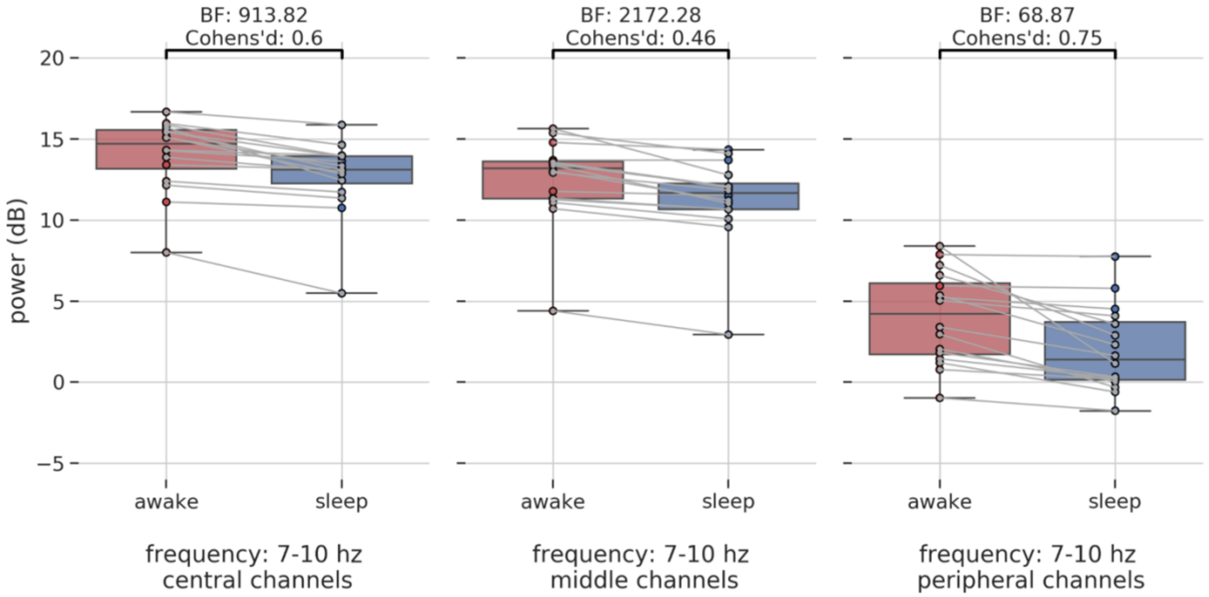

Supplementary Figure 6: Power differences across central, middle, peripheral channels in the frequency bands of 7-10 Hz.

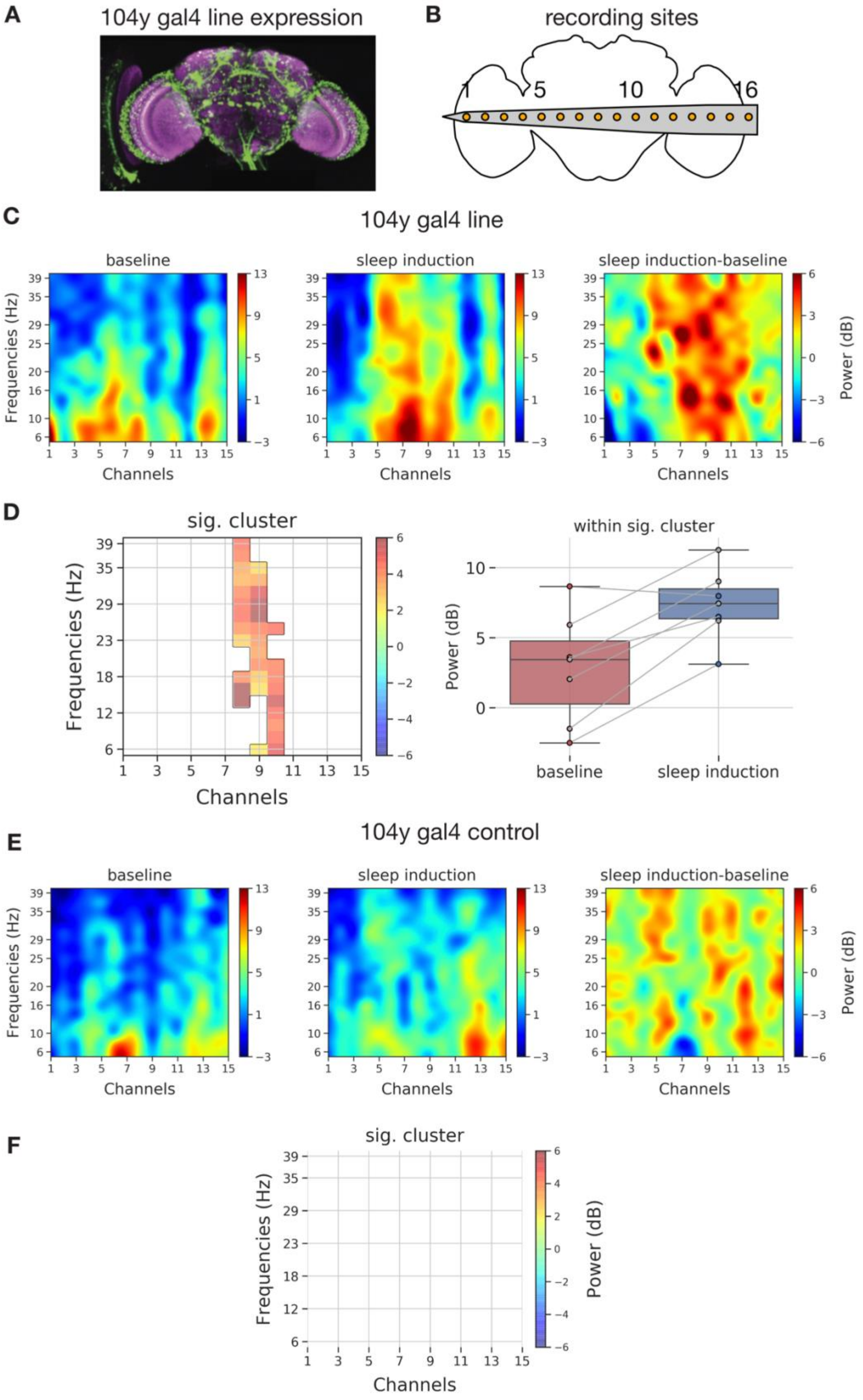
A
104y gal4 line expression
B
recording sites
1
5
10
16
C
104y gal4 line
baseline
sleep induction
sleep induction-baseline
Frequencies (Hz)
Channels
Power (dB)
D
sig. cluster
Frequencies (Hz)
Channels
within sig. cluster
Power (dB)
baseline
sleep induction
E
104y gal4 control
baseline
sleep induction
sleep induction-baseline
Frequencies (Hz)
Channels
Power (dB)
F
sig. cluster
Frequencies (Hz)
Channels
Power (dB)

Supplementary Figure 7: Spectral differences in thermogenetically induced sleep recorded using full brain probe. A) 104y-Gal4 pattern expressed by green fluorescent protein (GFP). B) electrode recording locations across the brain using full brain probe. C) Spectrograms of the 104y-Gal4 line across baseline and sleep induction periods and their differences. D) Clustering analysis reveals a single significant cluster mostly across central channels in all frequencies in the 104y-Gal4 line. E) Spectrograms of the 104y-Gal4 control line across baseline and sleep induction periods and their differences. F) Clustering analysis reveals no significant cluster in the 104y-Gal4 control line.

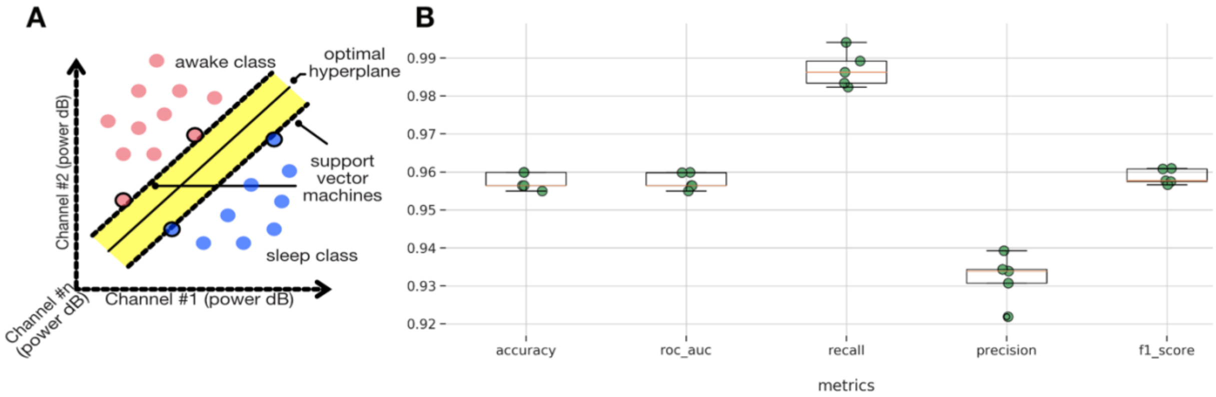

Supplementary Figure 8: SVM mechanics and metrics. A) Schematic indicating the optimal separation of awake and sleep classes using classifiers based on support vector machines. B) SVM based classifier performance across different metrics based on 5 different train/test data splits.

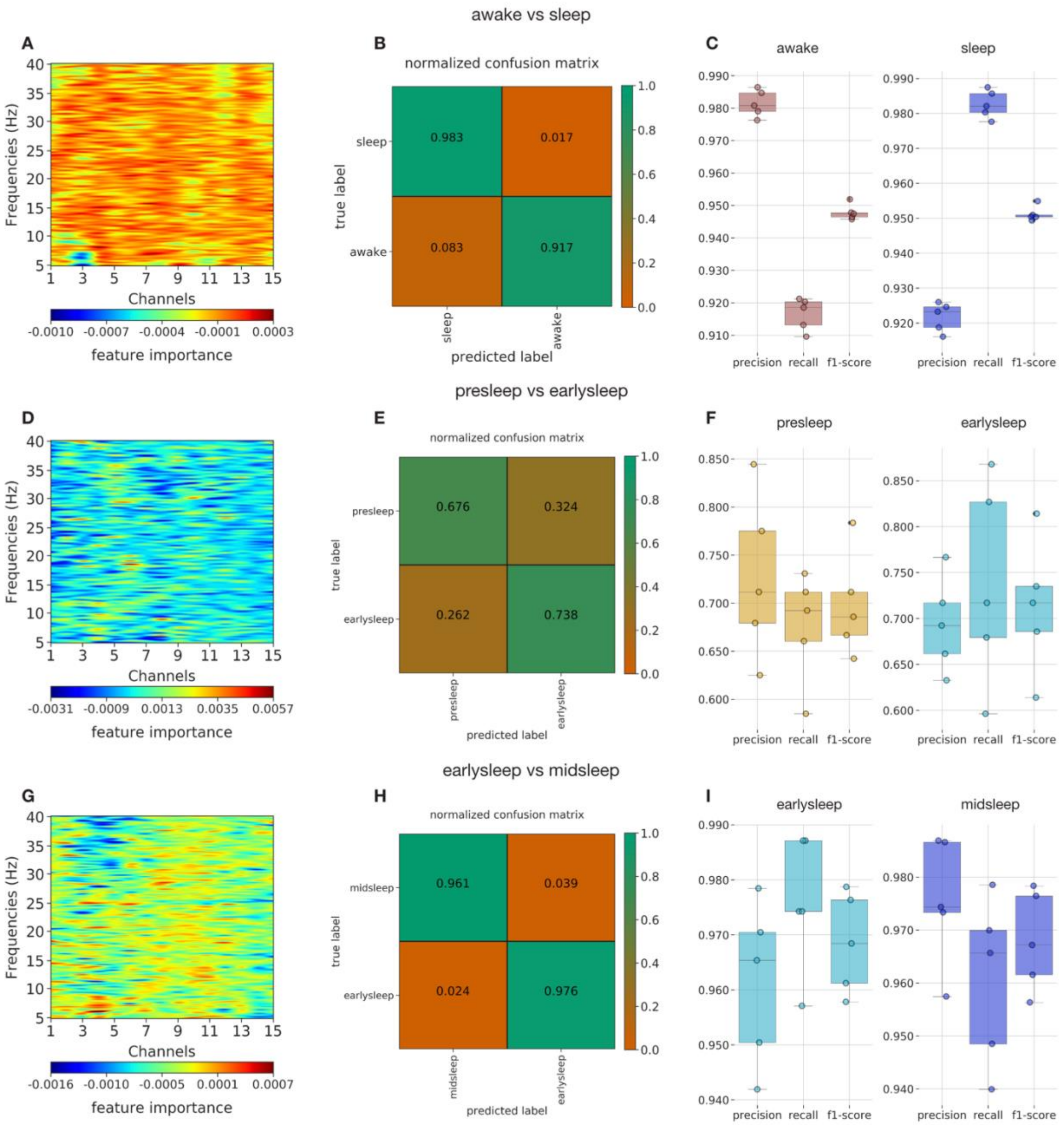
awake vs sleep
A
B
normalized confusion matrix
C
awake
sleep
Frequencies (Hz)
Channels
feature importance
true label
predicted label
precision
recall
f1-score
presleep vs earlysleep
D
E
F
presleep
earlysleep
earlysleep vs midsleep
G
H
I
midsleep

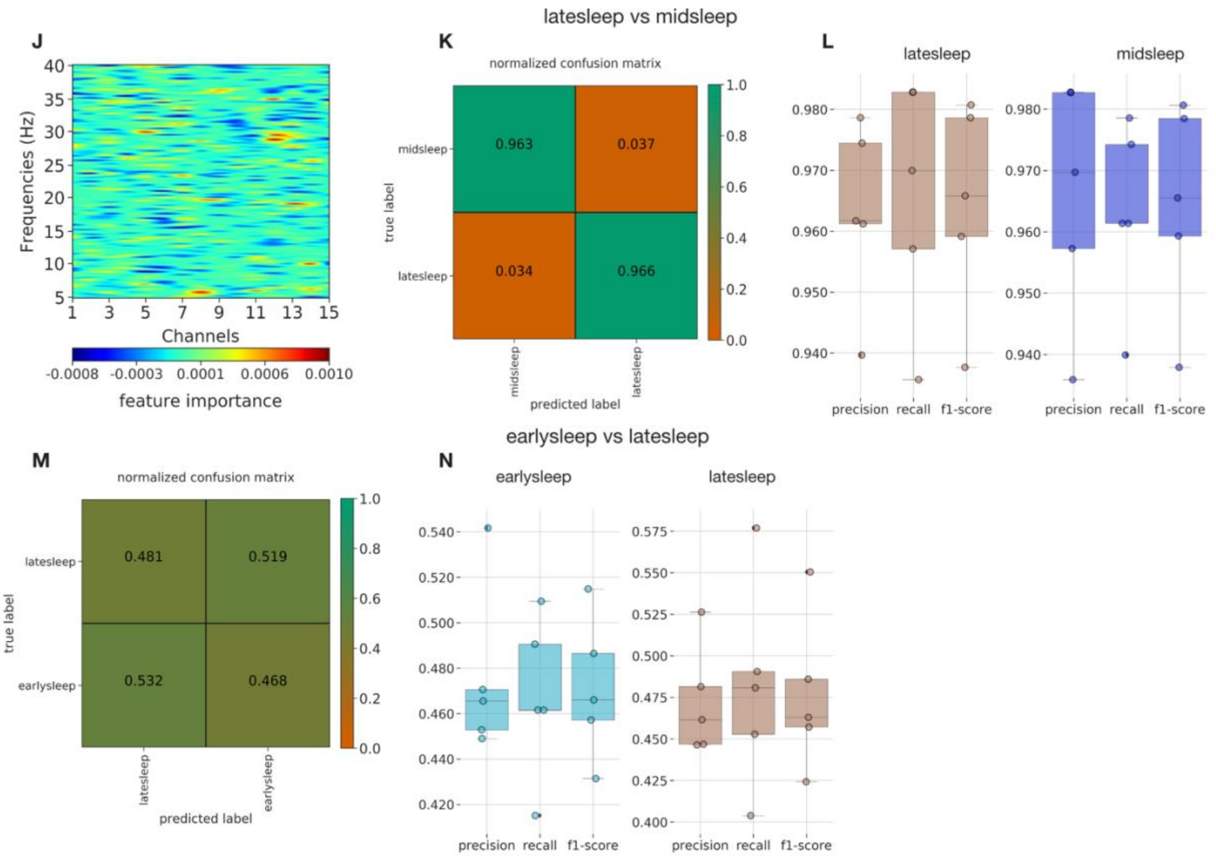

Supplementary Figure 9: Random forest classifier performance across different sub classes. Random forest classifier was trained to differentiate across awake and sleep periods: A) Feature importance reveals an ROI across all channels and almost all frequency bands as critically important. This cross validates the differences in the power spectrum across awake and sleep as shown in Figure 4D- left panel. B) Normalized confusion matrix. C) Performance metrics like precision, recall, f1-score. Random forest classifier was trained to differentiate across presleep and earlysleep periods: D) Feature importance. E) Normalized confusion matrix. F) Performance metrics like precision, recall, f1-score. Random forest classifier was trained to differentiate across earlysleep and midsleep periods: G) Feature importance. H) Normalized confusion matrix. I) Performance metrics like precision, recall, f1-score. Random forest classifier was trained to differentiate across latesleep and midsleep periods: J) Feature importance. K) Normalized confusion matrix. L) Performance metrics like precision, recall, f1-score. Random forest classifier was trained to differentiate across earlysleep and latesleep periods: M) Normalized confusion matrix. N) Performance metrics like precision, recall, f1-score.
